# Conversational agents enhance women's contribution in online debates

**DOI:** 10.1038/s41598-023-41703-3

**Published:** 2023-09-04

**Authors:** Rafik Hadfi, Shun Okuhara, Jawad Haqbeen, Sofia Sahab, Susumu Ohnuma, Takayuki Ito

**Affiliations:** 1 Department of Social Informatics, Kyoto University, Kyoto, Japan; 2 Graduate School of Engineering, Mie University, Tsu, Mie Japan; 3 Department of Behavioral Science, Hokkaido University, Sapporo, Japan

**Keywords:** Computational science, Information technology, Psychology and behaviour, Sustainability

## Abstract

The advent of Artificial Intelligence (AI) is fostering the development of innovative methods of communication and collaboration. Integrating AI into Information and Communication Technologies (ICTs) is now ushering in an era of social progress that has the potential to empower marginalized groups. This transformation paves the way to a digital inclusion that could qualitatively empower the online presence of women, particularly in conservative and male-dominated regions. To explore this possibility, we investigated the effect of integrating conversational agents into online debates encompassing 240 Afghans discussing the fall of Kabul in August 2021. We found that the agent leads to quantitative differences in how both genders contribute to the debate by raising issues, presenting ideas, and articulating arguments. We also found increased ideation and reduced inhibition for both genders, particularly females, when interacting exclusively with other females or the agent. The enabling character of the conversational agent reveals an apparatus that could empower women and increase their agency on online platforms.

## Introduction

There have been significant advances in women’s rights over the past decades, as many countries are constitutionally guaranteeing equal rights to all genders^1^. This progress remains, however, challenged in places where women have restricted access to services, employment, and governance^2^.

Women’s fight against discrimination has not always been easy and has gone from nonviolent movements and campaigns^3,4^ to protests and revolutions^5–7^. The recent demonstrations inside Iran have galvanized the world following the death of 22-year-old Mahsa Amini, who was arrested for allegedly violating the country’s mandatory veiling laws^8^. Iranian women are bravely protesting against her death but also an increasingly repressive regime targeting women, minorities, activists, and reporters^5,8^. Before the Iranian protests started, Afghan women had also taken to the streets of Herat, Kabul, Mazar, and Nimruz to protest against the Taliban since they took the country back in August 2021 and started reinforcing Sharia law^9^.

There seems to be no quick and easy solution to eliminating the centuries-long cultural obstacles to gender equality. Such equality is particularly challenging in societies with traditions restricting female participation in social, economic, and political activities. This situation persists despite the accumulating evidence that encouraging female participation in local governance redresses political and social gender imbalance and increases women’s involvement in income generation^2^.

Many factors still hinder female contribution to society. The effectiveness of legislative actions to empower women is often constrained by cultural attitudes that dictate stereotypical gender roles^10^. An alternative way to empower women is to therefore go beyond traditional boundaries. Information and Communications Technologies (ICTs), for instance, are now bringing communities closer to each other through regular and systematic information exchange^11–13^. The combination of ICTs and Artificial Intelligence (AI) is revolutionizing communication practices and how information is created, disseminated, and accessed. This transformation impacts how we communicate and affects the nature of the partners with whom we communicate. Online platforms now allow humans to communicate with *conversational agents*, chatbots, or social bots. Such agents can moderate online discussions and lead to improved deliberation and consensus^14–16^. With these abilities, AI-powered social networks and platforms are set to become the next-generation apparatus for democratic citizenry^17^.

Drawing on the participatory impact of women on group performances^18^, we claim that conversational AI can amplify women's presence and agency on social networks. To support this claim, we investigate two hypotheses. (Hypothesis 1) There are quantitative differences in how female and male debaters raise problems, propose ideas, and defend them on online platforms. (Hypothesis 2) Introducing a conversational agent that interacts with female and male debaters affects the quantity of the produced issues, ideas, and arguments. To test our hypotheses, we built a conversational agent and deployed it with Afghan citizens debating the fall of Kabul in August 2021. The agent engaged in the discussions alongside the participants and encouraged them by responding to their messages in various patterns. We then quantified the output of the participants based on the interaction patterns, the textual diversity of the content, and the number of issues, ideas, and arguments.

The present line of research addresses three pressing issues currently affecting the world. The first issue pertains to the rapid transformation of society with the advent of cutting-edge and controversial AI technologies such as ChatGPT^19^. The second issue is the persistence of gender inequality and the alarming discrimination against women, notably in countries such as Iran or Afghanistan^8^. The third issue is the fall of Kabul to the Taliban in August 2021 and how it intensified the oppression of women and further undermined the democratic transition in Afghanistan^9^. Our contribution is threefold. First, we elucidate gender differences in AI-powered online debates. Second, we show that conversational AI could amplify the contribution of women. Finally, we provide a principled socio-technical methodology that balances randomized controlled trials (RCTs), ICTs, and AI. Throughout this research, we do not address the political underpinnings of the events that led to August 2021, nor do we analyze the opinions expressed by the participants of our experiments.

In the following sections, we start by covering the impacts of ICTs on society within and outside Afghanistan. Then, we lay out the design of the study. We then provide the study method, which relies on an AI-powered online platform for social experimentation. We then provide the results, discuss the findings, and conclude the research with future perspectives.

## Can AI improve gender equality in Afghanistan?

### Social impact of AI

Online platforms are becoming an integral element of society for their proven ability to enhance human decisions and instigate social change^20^. They are used in medical education^21^, mental health care^22^, e-learning^23^, scientific inquiry^24^, city planning^25,26^, ecology^27^, political participation^20,28^, democratic deliberation^17^, and implementing Sustainable Development Goals (SDGs)^29–32^.

Despite their wide range of social applicability, using online platforms for large-scale discussions is still challenging. First, people often contribute unevenly when trying to shape the outcomes of a debate. There is a constant need for additional time to raise issues, develop ideas, or receive feedback. Finally, discussion threads on online forums are generally lengthy and unorganized. These factors render decisional processes on online platforms challenging, especially when the topics of the discussions are complex. Using ICTs could improve the quality of the discussions and facilitate decision-making. One could rely, for instance, on intelligent conversational agents for their ability to converse with humans. A conversational agent is a computer program that can reasonably converse with humans, albeit with limited and specialized capabilities. Such agents could, for instance, moderate group discussions by managing the discussion time, encouraging members to participate evenly, and organizing their opinions^33^. Conversational agents could interact with stakeholders to collect ideas that satisfy conflicting needs and to consider opposing viewpoints^34^.

The earlier versions of conversational agents relied on simple pattern matching when interacting with humans. With the recent prowess of Machine Learning (ML), intelligent agents are built with advanced techniques, such as Deep Learning and Large Language Models (LLMs)^19,35,36^. The conversational AI built by companies such as OpenAI and DeepMind can now produce meaningful text that seems to be reasonably coherent^19^. ChatGPT, for instance, has mesmerized the world with its ability to produce quasi-sound essays^19^. The methodology adopted in our study uses Deep Learning to classify the participants' messages and guide the agent's behavior. This approach allows the agent to respond to any given message to facilitate the discussions. In practice, the agent identifies argumentative discourse in a discussion, builds its semantic representation, and responds with adequate messages. This technique allows the agent to adapt to various users and discussion topics. Adopting rules to generate natural language imposes constraints on the agent and reduces any risks of producing meaningless or harmful content^37^. In addition, the argumentative nature of the discussions must drive the generated utterances of the agent. We, therefore, adopt a formalism often used in debates, termed the Issue-Based Information System (IBIS)^38^. IBIS can help identify, structure, and settle wicked problems by providing information pertinent to the discourse. At its core, the IBIS formalism deconstructs any discussion into issues, ideas, and arguments (pros and cons).

### Importance of ICT to gender equality

The *smartification* of society using Information and Communication Technologies (ICTs) has revolutionized several aspects of our daily lives. Socio-technological systems are becoming preferred over conventional human-assisted intervention. The consequent growth of Information and Communication Technologies for Development (ICT4D) has also helped deploy systems that facilitate social development. A surfeit of studies has piloted these platforms to address the effect of digitalization and *algorithmification* on society^25,39^. For instance, the diffusion of ICT4D provides more possibilities to empower women by enhancing gender-based digital equality^11–13^.

Despite decades-long fights against gender disparities, inequalities still exist worldwide but with varying degrees and are more prevalent in conservative and male-dominated countries^40,41^. The causes, factors, and outcomes were addressed in various studies using conventional tools and ICTs^42^. Governments have adapted ICT4D into their policies to empower citizens^43^. Researchers have also conducted feminist science and technology studies to propose ways to empower women's social presence using ICT4D^44,45^. For example, some studies show that ICT can contribute to economic well-being and empower women's community building and political organization^46^. ICT can make women's voices heard through the role of digital networks in feminist movements^47^. In sum, ICT is a powerful tool for women to overcome discrimination, achieve equality and well-being, and participate in the decisions that determine their lives and the future of their communities^11–13^. However, this potential to empower women in online communities requires additional technical support in economically disadvantaged and technologically underdeveloped regions. Online forums, in particular, are perceived and used differently in conservative, male-dominated, and war-ravaged countries such as Afghanistan, which might lead to a digital divide^48^. Priority should therefore be given to bridging this divide and aiming at more inclusion in online communities. At the same rate of proliferation of ICTs and with the increasing abundance of data, social networks are gradually relying on chatbots^49^. Researchers are experimentally examining how humans interact with such entities in online discussions^25,39,49^.

In our study, we highlight the case of Afghanistan for its importance in drawing on the research gap in ICT4D in empowering marginalized communities, particularly women. Numerous factors impose limits on the social presence of Afghan women despite the evidence showing how ICT4D can benefit them and enhance their sense of agency^50^. We, therefore, propose to investigate gender differences using ICTs to pave the way to the kind of digital inclusion that could qualitatively empower their online presence. We particularly rely on conversational agents with linguistic and social capabilities. Such agents will interact with Afghan citizens in randomized, controlled online experiments. To the best of our knowledge, no research has shown thus far how AI-assisted forums could empower women in war-torn, conservative, and male-dominated countries such as Afghanistan. In the next section, we start by laying out the demographic, social, and political context in which we conducted our study. Factors such as the literacy rate, age, and Internet reach are crucial in assessing the effectiveness of ICT.

### The fall of Kabul in August 2021

Given its social and cultural characteristics, Afghanistan constituted an adequate testbed for our study. It is a landlocked country of approximately 250,000 square miles located at the crossroads of south-central Asia. The country is ethnically, linguistically, and religiously diverse, with roughly 38 million, half women, and 26.6% living in urban areas^51^. The country has one of the youngest populations in the world, with nearly two-thirds of Afghans under 25 years of age. The literacy rate in the country is 37.27%, with 23% for women and 52% for males as of 2021. The internet reach in Afghanistan is 18% as of 2020.

Many Afghans had to escape the country in August 2021, making finding participants difficult. Migration in Afghanistan has for decades been a major social force, driven mainly by war, political turmoil, and socioeconomic inequities^52^. Emigration dramatically increased during the Soviet occupation (1979–1989), Afghanistan’s post-Soviet civil war (1989–1992), the Taliban regime (1996–2001), the US war in Afghanistan (2001–2021), and the return of the Taliban to power in August 2021^53^. These waves of war and political instability resulted in five mass movements of refugees and immigrants. The first wave of refugee outflow emerged during the Soviet invasion and its withdrawal in 1989. Over 6 million Afghans fled, marking the fleeing height within Afghanistan’s long migration history^52^. The second wave had significant internal displacement during Afghanistan’s post-Soviet civil war. The third wave of refugee outflows emerged during the Taliban regime, with more than 1.2 million fleeing the country. The fourth wave of refugee outflows occurred during the war in Afghanistan, with more than 0.9 million fleeing the country^52^. The aftermath of the US military departure from Afghanistan after 20 years of war has marked the fifth wave created a refugee crisis that displaced more than half a million.

The Taliban seizure of power in August 2021 has intensified Afghanistan's humanitarian crisis. The country was already confronting acute food insecurity and severe drought, followed by the COVID-19 pandemic. The public health infrastructure faced logistic challenges, given that the Taliban campaign displaced more than half a million people^54^. The Taliban advance has brought about a public health catastrophe touching the most fragile members of society^54^. Afghan students suffered from mental health deterioration with an alarming prevalence of PTSD symptoms, depression, and suicidality^55^. Afghan women are losing fundamental rights under the authoritarian regime of the Taliban, with Burqas becoming mandatory and access to public facilities and education being curtailed^56,57^. Amid this gradual deterioration of Afghan women's rights, our study attempts to take their struggle to the online sphere, hoping that AI could contribute to their emancipation and support their voices on online platforms.

Returning to our original question, “*Can AI Improve Gender Equality in Afghanistan?*” leads us to believe that while the potential exists, it may be limited in scope. ICT is insufficient to address the deeply rooted societal issues faced by Afghan women. Systemic changes are required in various aspects of society, such as education, legal protection, and political representation. While AI-enabled platforms may play a role in mediating decisions and instigating social change, they should be approached with a balanced perspective, acknowledging their limitations, benefits and risks.

## Methods

### Research design

**Figure 1:**
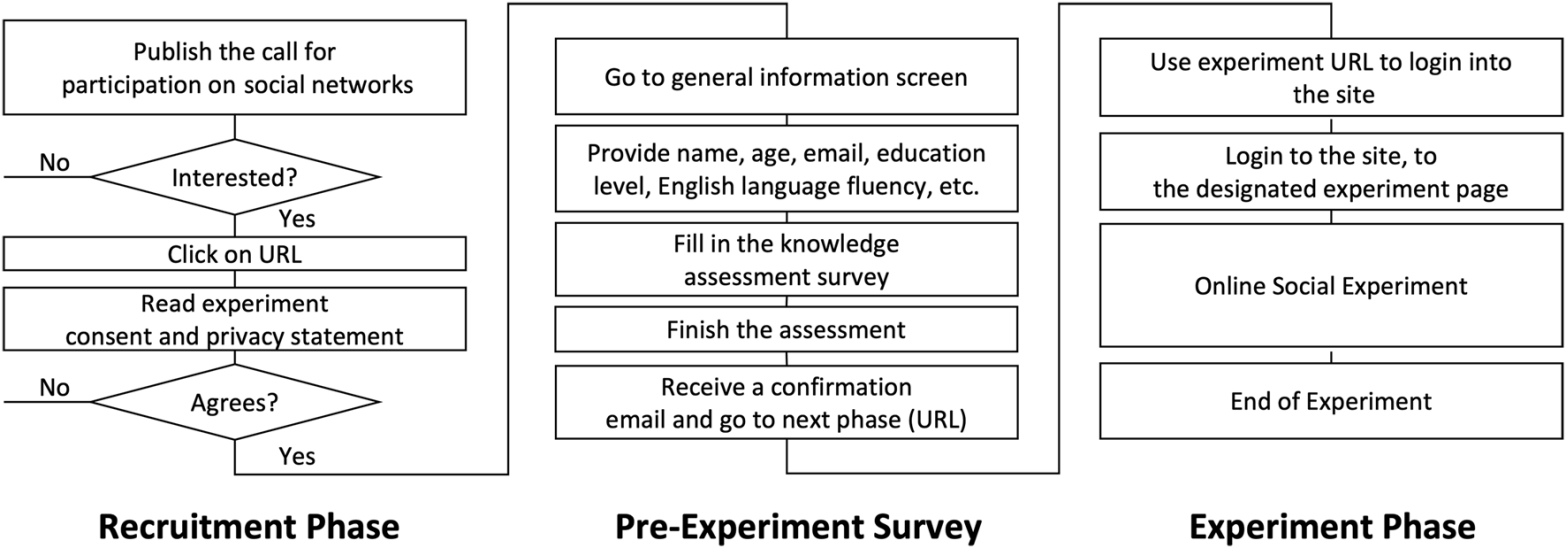
Workflow of the study from recruitment to the online experiment with n = 240 subjects.

We adopted a stratified random sampling strategy that assigns the n = 240 subjects to randomized controlled trials (RCTs) that either receive the intervention of the conversational agent or do not. We started by recruiting a quasi-random sample of n = 10,000 Afghan citizens who answered our Survey Monkey call and filled out the selection questionnaire. Based on our selection criteria (Age, Education level, English skills, ICT skills), we selected n = 5898, eligible participants (See supplementary material S4 for the distributions). We then gave them knowledge evaluation questionnaires. We finally chose n = 240 participants based on their scores, proven ability to communicate in English, and use of social networks. Figure 1 illustrates the recruitment process up to the online experiment. Each subject was rewarded 12.5 USD for their participation in the 2-h discussion to which they were randomly assigned. The study protocol was approved by the Research Ethics Committee of the Graduate School of Informatics at Kyoto University (Reference KUIS-EAR-2021–020). All experiments were conducted in accordance with the approved protocol, which met the requirements of the Declaration of Helsinki. All participants provided written informed consent before participating in the experiments.

**Figure 2:**
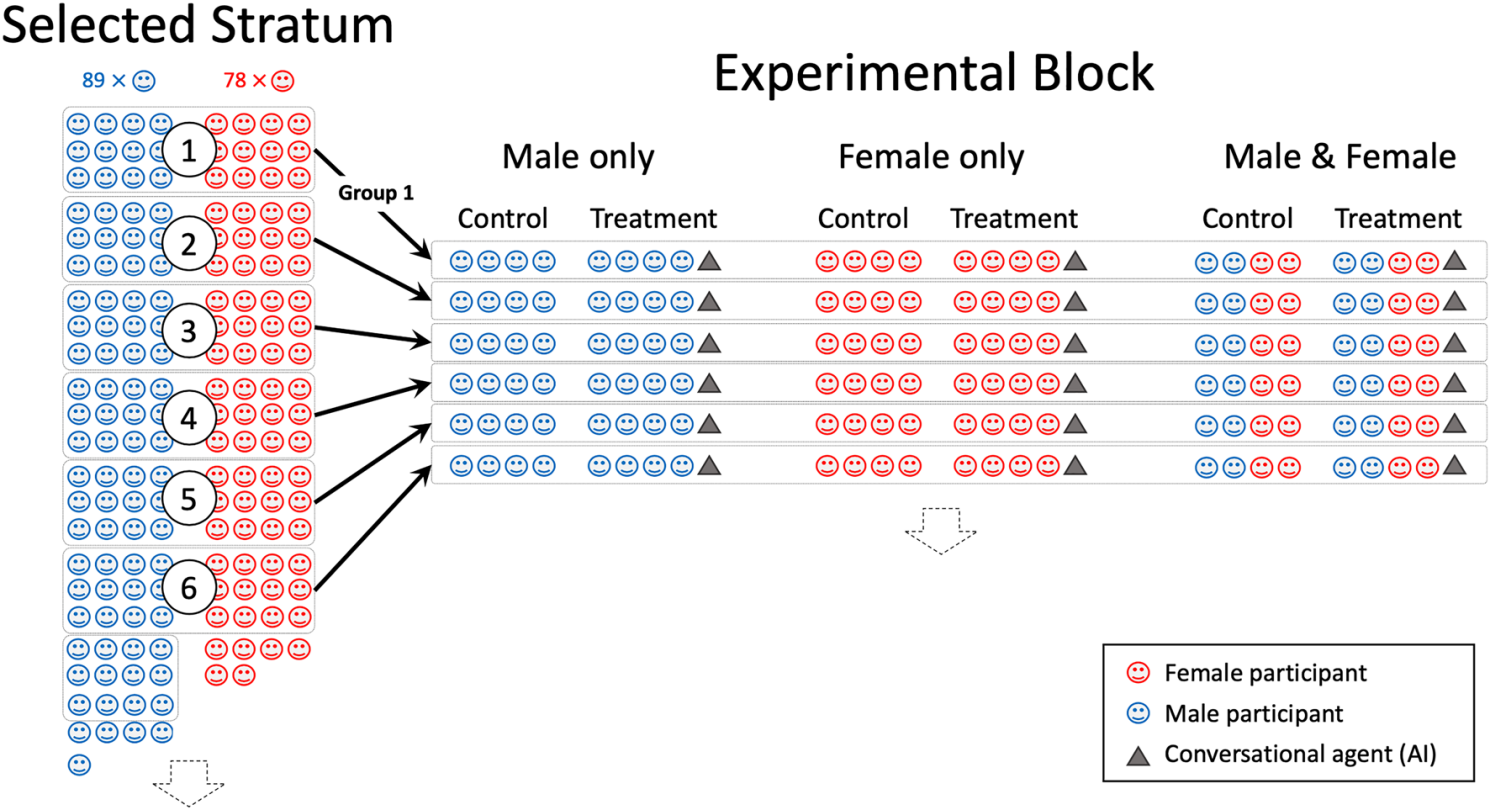
When constructing the experimental groups for the discussions, we start with a stratum of, for example, n = 167 participants that satisfy the selection criteria. An experimental block is then generated, containing six independent groups containing n = 24 participants each. Each group has three mixtures, with control and treatment (presence or absence of the agent) subgroups of n = 4 participants each.

To investigate our hypotheses, we looked at groups composed solely of females, groups composed solely of males, and groups that are a mixture of the two. The groups were exposed to controls where there is no conversational agent to interact with and treatments where there is an agent to converse with according to different patterns of interaction. This design allowed us to identify the agent's effect on the participants. Out of 240 participants, we formed one experimental block, as shown in Fig. 2. The block was divided into male-only, female-only, and mixed groups of participants.

The experimental groups in the example of Fig. 2 were formed using a uniform selection of the participants into control and treatment groups based on gender, with an even distribution of males and females out of the original pool. The final n = 240 subjects of the experiments were within the 20–30 age range; had intermediate to upper-intermediate English language capabilities, and had college, graduate or postgraduate education. The knowledge questionnaire evaluated their knowledge on themes covering Afghan society, education system, gender equality, economics, and politics. The choice of the number of participants per subgroup n = 4 accounts for the specificities of online discussions. Large groups in online experiments are generally challenging to control^18,58^, particularly in the presence of AI. In addition, there are other constraints to this number, such as the cases where users cannot see the content when there are too many posts. These constraints justified having four participants within one single discussion subgroup.

### The conversational agent

The agent relies on a *Natural Language Processing (NLP) engine* that extracts, processes, and produces argumentative text. The details of the overall system are provided in the Supplementary Material (S1). The agent first extracts the utterances from the raw textual content of the discussion. The content is then classified into issues (or questions), positions (or ideas), or arguments (pros and cons) according to the issue-based information system (IBIS)^38^. IBIS is a discourse template that guides the identification, structuring, and settling of wicked problems. Being compact and intuitive, this model could be implemented using Deep Learning techniques and trained to classify argumentative text. The used implementation relies on an Artificial Neural Network (ANN) called Bidirectional Long Short-Term Memory (Bi-LSTM) to classify the textual content into IBIS categories^39^. Since the discussions we focus on are argumentative, the agent must be capable of identifying argumentative content to facilitate the debates meaningfully. To this end, the agent transforms any IBIS representation into an argumentation graph and reasons about its elements using an argumentation engine.

The agent's identity is not disclosed to the participants beforehand. The agent interacts with the participants on the basis of their characteristics by following predefined facilitation rules. The agent will wait for the participants to post their messages, and once it identifies ideas, issues, or arguments, it will reply with a facilitation message that either asks the participant to elaborate on an argument, to back up an idea with an argument, and so forth. An example of such mechanism is provided in the supplementary material S1.2. Moreover, the agent behaves neutrally in all discussions except in the mixture cases, where it tends to react more positively to women's posts. Behaving favorably towards women's contributions will help identifying whether it is possible to artificially counteracting the potential inhibitions that women might experience in the presence of men or not. Moreover, it helps in investigating whether supportive agent-based mediation could translate to enhanced contribution or not.

In the experiments, the four participants of each subgroup are anonymously identified using prefixes “Mr. A,” “Mr. B,” “Mr. C,” and “Mr. D” for men and “Ms. A,” “Ms. B,” “Ms. C,” “Ms. D” for women to distinguish between their genders.

### Discussion topic

The choice of the topic of the study is crucial in the sense that it should steer the debates. Examples of such topics could include polarizing issues on religion, politics, or governance^2^. Our selected topic had, in a way, to reflect the ongoing social and political turmoil in Afghanistan. The discussion topic was posted as follows (see S1.2 for more details).

Dear Afghan fellows,

Thank you for participating to discuss using D-Agree.

In August 2021, the provincial capitals in Afghanistan and Kabul fell into the hands of the Islamic Taliban movement. For many Afghans within Afghanistan and in the diaspora, the distress is palpable as they face uncertainty about the country's future.

How did we get here? What will happen next? Do you think that the Taliban-led government will be recognized as a legitimate regime by the Afghan people and the international community?

We invite you to have a discussion about the topic by providing your opinions, ideas, and supporting them with arguments only in English.

### Measures

**Figure 3:**
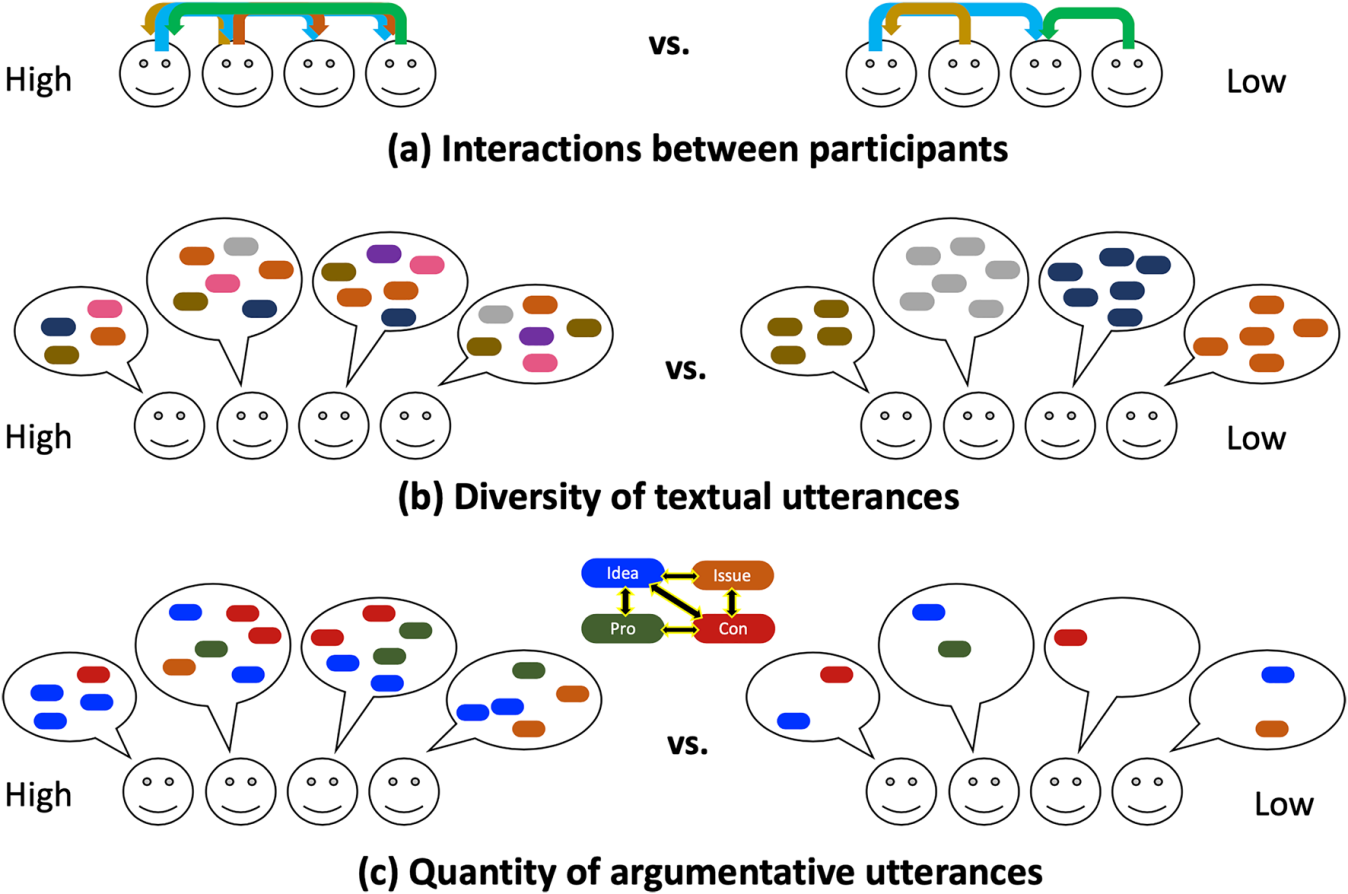
Interactional, textual, and argumentative views of the discussions.

There are several ways to evaluate participants' output in an online discussion. Within a group, it is possible to look at the average member's knowledge and maximum member intelligence^58^. Other metrics include social sensitivity using the “reading the mind in the eyes” test^58^. In online discussions, there are metrics on opinion diversity and the evenness of the contribution^33^. Some metrics do not account for the content but look at the temporal dynamics of the interactions^59^. Herein, we adopt measures with three levels of interpretation, as shown in Fig. 3. The first level looks at the interactions between the users without accounting for the content of the messages they exchange^59^. The second level looks at the diversity of the used vocabulary. The third level looks at the argumentative semantics of the messages by automatically classifying the content into one of the four IBIS categories^38^.

The first level of analysis, in Fig. 3a, identifies the patterns of interaction between two groups of participants, with one group (left) exchanging messages more than the other (right). In our study, interactions refer to the dynamic exchange of responses between participants, encompassing both the sequence of replies and the frequency of responses. We are particularly interested in numbers of responses between the different actors. Examples of such interaction patterns are provided in Supplementary Material S1.2. In Fig. 3b, we examine the textual diversity, or uniqueness, of the employed vocabulary. In Fig. 3c, we compare the groups based on the number of the IBIS elements identified as issues, ideas, and arguments (pros or cons). The various measurements were performed on distinct samples representing different experimental subgroups composed of n = 4 participants each.

### Informed consent

Informed consent was obtained from all the participants of the study.

## Results

We performed ANOVA tests to compare the means of the measures for different groups and gender compositions. Before the ANOVA, we conducted normality tests to assess the distributions of the measures. The detailed figures of the tests are available in the supplementary material (S2 and S3). In the following, we adopt a notation where the symbol ◯ represents female participants, ▢ represents male participants, and △ refers the conversational agent present in each discussion alongside the human participants. We start by showing the patterns of interaction between the participants, regardless of the content. This approach is commonly used to analyze the patterns of verbal interactions in couples^59^.

### Interaction between participants

**Figure 4:**
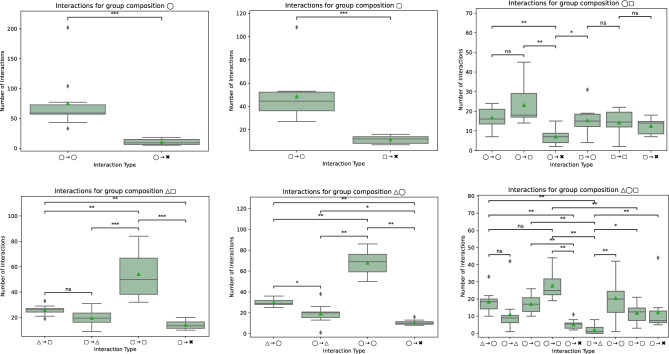
Interactions between female (◯) and male (▢) participants across different group compositions. The cross symbol (✖) denotes the startup message that sets the topic of the discussion at the top of each discussion thread.

Patterns of interaction between the participants are shown in Fig. 4.

From the interaction counts, participants are more interested in directly engaging with each other by replying to each other’s messages than engaging with the conversational agent or posting under the root post of the discussion. Secondly, female participants tend to be more engaged with each other across different group compositions. Finally, in the presence of the conversational agent that uniquely addresses female participants (△◯▢), women tend to be engaged more with other female and male participants.

### Content diversity

The textual content produced by the participants was assessed with respect to its uniqueness within the context of each discussion.

**Figure 5:**
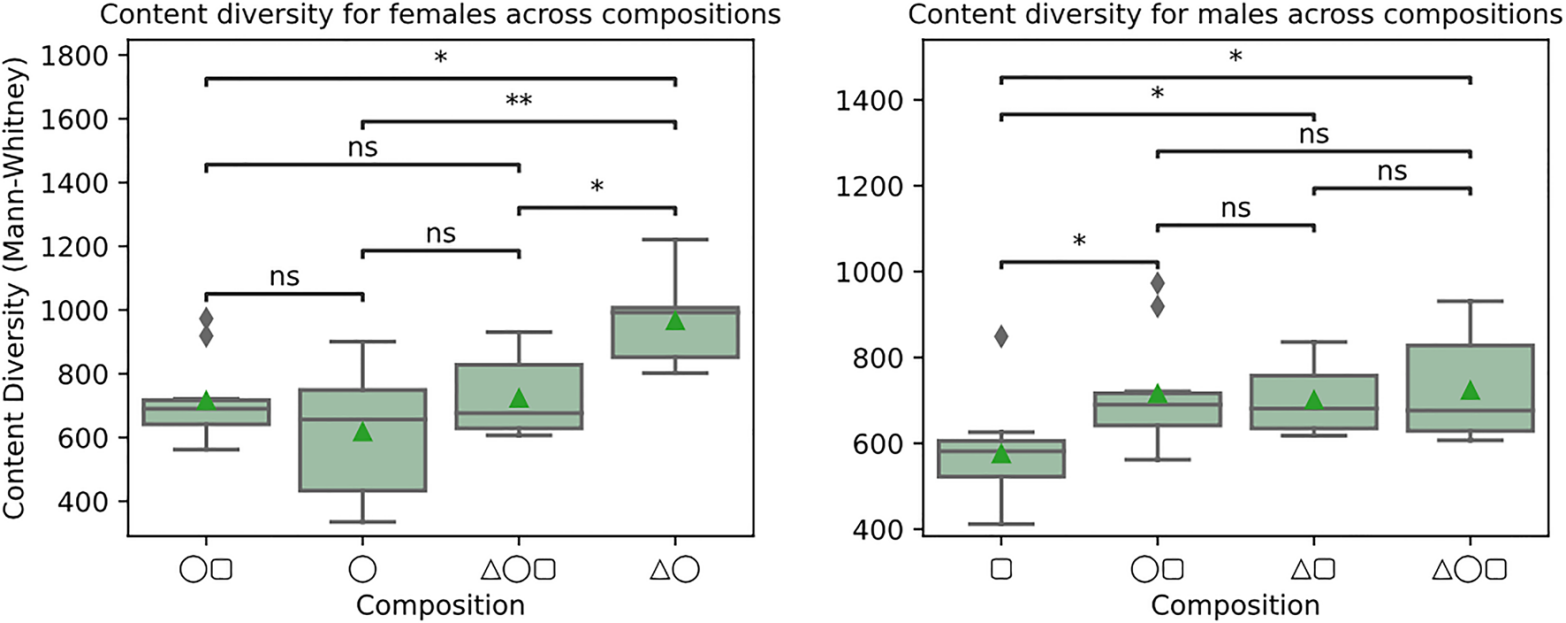
Content diversity for female (◯) and male (▢) participants across different group compositions with and without the conversational agent (△).

Figure 5 shows such quantities for female and male participants in different group compositions. Female participants tend to produce more diverse content when taken in isolation with the conversational agent than when alone (p < 0.01, △◯ > ◯), with male participants (p < 0.05, △◯ > ◯▢), or with both (p < 0.05, △◯ > △◯▢). Male participants also tend to produce relatively more diverse content in the presence of the conversational agent (p < 0.05, △▢ > ▢) or female participants (p < 0.05, ◯▢ > ▢) than when alone.

Finally, we look at whether there are differences between the content produced by both genders (hypothesis 1). First, we look at the quantities of one particular kind of IBIS utterance across different group compositions. Second, we look at the distribution of all IBIS utterances within each group composition. These two levels of analysis leverage the variability across IBIS content and the heterogeneity of the groups. To test whether a conversational agent has a quantitative effect on the produced content or not (hypothesis 2), we look at the variability in the number of issues, ideas, supporting arguments (pros), and attacking arguments (cons) across compositions with different mixtures of human participants and the conversational agent.

### IBIS across group compositions

**Figure 6:**
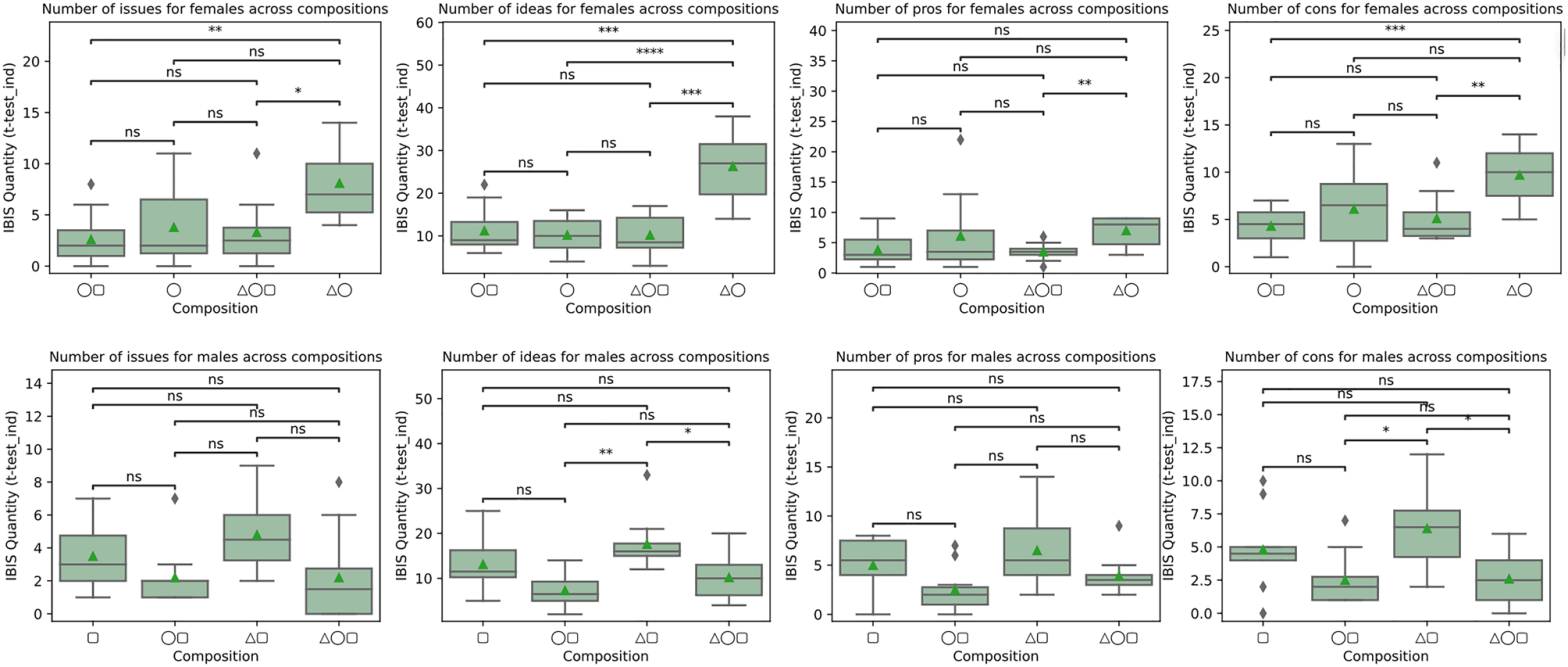
Prevalence of IBIS utterances for female (◯) and male (▢) participants across different group compositions with and without the conversational agent (△).

The prevalence of IBIS utterances (issues, ideas, pros, and cons) across different group compositions is shown in Fig. 6. We start by looking at the performance of the female participants across all group compositions in the upper part of Fig. 6.

Female participants tend to raise more *issues* when in isolation with the conversational agent than male participants (p < 0.01, △◯ > ◯▢). Similarly, the conversational agent makes a clear difference regarding *idea* generation when interacting in isolation with women (△◯ > ◯, ◯▢, △◯▢). This is particularly the case when comparing the number of ideas generated by the female participants alone and with the conversational agent (p < 0.0001, △◯ > ◯). In terms of the supportive arguments (*pros*), female participants tend to provide more arguments when in isolation with the conversational agents than with the presence of both male participants and conversational agents (p < 0.01, △◯ > △◯▢). For raising attacking arguments (*cons*), the female participants tend to provide more cons with the conversational agent in isolation (p < 0.001, △◯ > ◯▢) than with male participants in isolation and with the conversational agent (p < 0.01, △◯ > △◯▢). The performance of the male participants across all group compositions is shown in the lower part of Fig. 6. There is no clear significance in the prevalence of *issues* or supportive arguments (*pros*) across different group compositions, with or without the conversational agent. Male participants tend to provide more *ideas* in the presence of the conversational agent (p < 0.01, △▢ > ◯▢). Similarly, they tend to raise more cons when they are solely in the presence of the AI (p < 0.05).

### IBIS within group compositions

**Figure 7:**
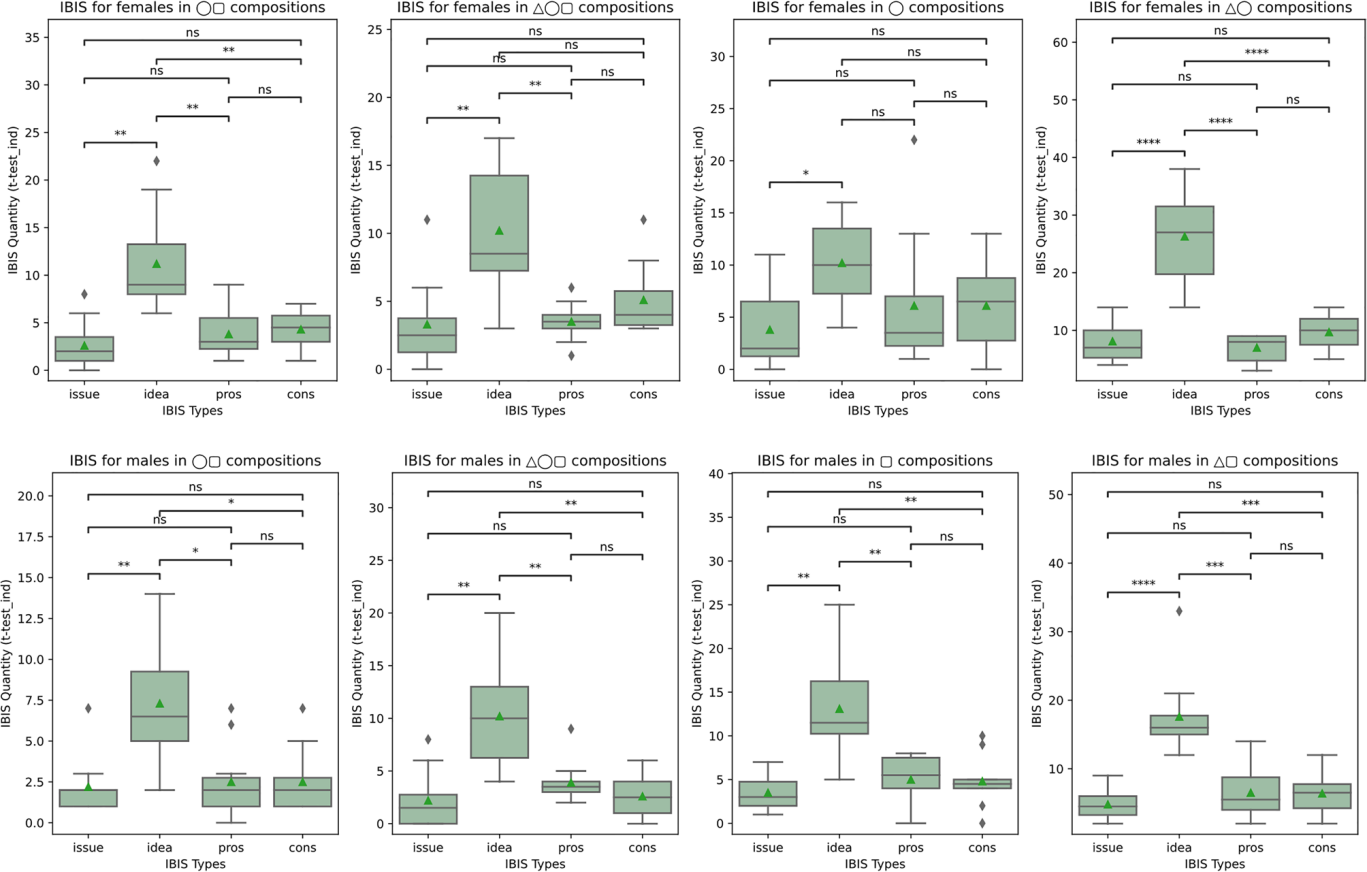
Prevalence of IBIS utterances within group compositions for female (◯) and male (▢) participants with and without the conversational agent (△).

For the IBIS elements within each group composition, we obtain the results in Fig. 7.

We start by looking at the quantity of IBIS utterances of the female participants across all group compositions in the upper part of Fig. 7. Female participants generate more ideas than issues, cons, and pros, particularly when taken with the conversational agent in isolation (Fig. 7, top, p < 0.0001 △◯). Similarly, male participants provide more ideas across all compositions (Fig. 7, bottom).

## Discussion

### Statement of principal findings

From the quantities of the raised issues, ideas, and arguments, female participants tend to be more active in the absence of male participants. The production of ideas is particularly intensified in the presence of the conversational agent, as shown at the top of Fig. 6 (p < 0.0001, △◯ > ◯). This is partly due to the differences between genders in face-to-face and Web-based discussions^60^. Men tend to over-proportionally speak face-to-face, whereas women over-proportionally post messages in Web-based settings^60^. This suggests that women prefer written communication more than men or written communication over spoken communication.

Other than the increase in the production of ideas, the same could be said for another type of utterances in the argumentative discourse. That is, raising issues, problems, or questions generally indicates the lack of something, or a gap between the current situation and its idealization, suggesting that something needs attention, clarification, or resolution. When coming from women, such utterances are often inhibited and rarely reported in some social contexts. The inhibition would cause a reduction of the contribution of the female participants in the presence of male participants and is often linked to opinion inhibition under social pressure akin to some cultures^41,61^. Such suppression phenomena are often attributed, for instance, to religion, education, and status^62^. The same phenomenon is also observed in the male participant contribution (Fig. 6, bottom), albeit weaker. Other factors that link to the suppression of opinions are also due to group sizes and disparity in subgroup power^63^. Such suppression is also more prevalent in the case of the production of issues and cons, which semantically and within the IBIS discourse of the experiments, are more related to “questioning” or “attacking” an established stance. In the context of a mixed-gender debate in the cultural setting of Afghanistan, this could be perceived as challenging patriarchal, stereotypical gender roles and expectations^10^.

Looking at the effect of the conversational agent in addition to the results of Fig. 6, when women are in isolation with the agent (△◯), it contributes to increasing the number of ideas, as shown in Fig. 7. This is corroborated by the vocabulary diversity used in the same setting, as shown in Fig. 5. The ideation resulting from this symbiosis indicates the role of AI in supporting human decision-making^16,28^ and shows that social interactions could be enhanced using AI beyond the existing gender divides.

### Implications of the research

Apart from the potential of AI, Hussain et al.^50^ emphasized that different types of ICTs can enhance Afghan women’s *agency* by advancing their economic, political, social, and physical conditions. In this case, the use of ICTs should not be perceived as a neutral act deprived of any political implication, but it should be understood in relation to its transformative power in society. That is, in relation to questions of changing the sense of self-worth of Afghan women, the range of accessible choices beyond imposed gender roles, and even the relationships in which they are embedded. It is, therefore, important to understand that true empowerment involves a *transformative agency* that allows women to challenge gender inequality directly^64^ as opposed to the existing *restricted agency* that continues to limit their choices^12^.

The potential of current ICTs in advancing gender inequality is still limited and is mostly due to the collective lack of awareness. It could therefore be enacted through the ongoing integration of AI in social networks, which primarily touches the younger population. This integration is inevitably changing the nature of the partners with whom humans interact on social networks, which could now work hand-in-hand with autonomous and intelligent agents^17^. Our results advance the possibility of adopting a *symbiotic agency* that integrates women, men, and artificial agents. It is necessary to emphasize the inclusiveness of this form of agency because of its potential to be extended to other religious, ethnic, and cultural minorities in Afghanistan. Finally, the collected opinions of the participants are partly the result of their symbiotic interaction with the conversational AI. The overall contributions might consist, in a sense, a collective perspective on the current situation in Afghanistan. This could prove valuable in informing policy makers on the diverse range of views and insights from the informed citizens, supporting them in making more informed decisions^16,25^ and formulating policies that address the complex and dynamic challenges facing the country.

### Limitations

We now discuss some limitations of our analysis as well as how these limitations can be addressed or mitigated to enhance the robustness of our findings.

First, our work comes with the inherent limitation on the demographic of the participants. Several factors constrained who could be part of the study, including the online medium utilized for the experiment, the minimum prerequisite for ICT skills, internet access, and English proficiency. The safety challenges at the time of the study further hindered our ability to target a bigger population. The study could, however, be expanded with a more representative demographic by implementing strategies that we have previously envisioned and tested in “hybrid” experiments conducted in Afghanistan^43^ and Japan^25^. We propose three several solutions to address the limitations. First, one could adopt the official language of Afghanistan (Dari) within the AI system. This implies implementing and training the conversational agent with Dari, which has more data than other local languages. Second, we could adopt practical solutions to address the Internet access limitations, such as installing Internet spots in Gozar-related gathering halls and allowing more participants in the online experiments. A “Gozar” is a traditional district unit in Afghanistan^65^. Third, we could adopt mediation techniques to involve less capable groups such as the illiterate, senior citizens, women, and children. Mediation was previously used in an experiment in the city of Kabul, where the discussions were facilitated by the Kabul municipality chief of staff, acting as a mediator that transcribed opinions from Dari to English on the AI system^32^. In another study^25^, human-mediated facilitation was adopted to gather opinions from senior Japanese citizens on next-generation planning. Augmenting the conversational agent with additional linguistic capabilities could help considerably expand the subjects pool beyond the English-speaking, educated, and ICT-literate population. Adopting one unifying language (Dari) is ideal since allowing multilingual discussions will complexify the study design and the implementation of the AI. In this case, the AI must be culturally aware of the nuances in the languages, which is currently a persistent technical challenge in AI research. Adopting mediation techniques to include diversified citizens necessitates redesigning the experiments to account for the mediating effects of the human actors and the conversational agent.

The second limitation to the study is language. Afghanistan is a multilingual country with uneven distribution of language proficiency among the population. Requiring the participants to be proficient in English is one solution. First, it ensured a clear and consistent understanding of the study instructions among all participants regardless of their native language(s). Secondly, it ensured effective communication with the agent, which as an AI system, was trained to understand the English language. Adopting the English language in the AI and the experiments allowed us to collect accurate data and minimize potential language-related confounds during data analysis. In addition, one possible effect of the English language is that it might encourage positive attitudes across different linguistic groups. Individuals who can speak the language of individuals from other language backgrounds can more easily engage in conversation with them, which may lead to more positive attitudes towards these individuals^66^.

Had the study been conducted in Dari or Pashto instead of English, there would likely be differences in the experimental design and AI system. The differences will pertain to the following aspects.

1. The AI will have to be implemented and trained to support additional languages, which is not necessarily an easy task due to the lack of textual data in languages such as Pashto. Moreover, embedding multilingualism in conversational systems is still a challenging domain of investigation.
2. The distributions of the target participants might differ based on the language chosen for the study. Conducting the study in Dari or Pashto would increase the demographic of potential subjects. For example, if the study were conducted in Dari, it might be easier to recruit participants from certain regions in Afghanistan where Dari is widely spoken.
3. Different languages in Afghanistan come with their own cultural nuances, sensitivities, and, most importantly, reactions to the fall of Kabul to the Taliban (who are predominantly Pashto speakers). The choice of the language can impact how this sensitive topic is framed and discussed. Whether training the agent to function in one or several languages, we must be aware of and adapt it to cultural differences to avoid topical misunderstandings. For example, Dari speakers are not as proficient in Pashto as Pashto speakers are in Dari. Therefore, the choice of language for the subjects and the conversational agent is crucial and should depend on the ability of the participants to write in that language and the ability of the conversational agent to apprehend it.
4. Participants might feel more forthcoming when responding in their native language. Conducting the study in Dari could potentially reduce response bias that may arise when participants are not as proficient in English and feel compelled to answer in a language, they are less comfortable with.
5. If the topic of the discussion is initially created in English and then translated into Dari or Pashto, the translation process could introduce errors or alter the intended meaning. To mitigate this, it would be crucial to use one unified language in the topic, discussions, and conversational agents.
6. Depending on the linguistic features of Dari or Pashto, some concepts or ideas may be easier to express in one language than the other depending on the complexity of the topic, the time allocated for the discussions, the sizes of the groups, etc.

Conducting the study in Dari or Pashto would involve tailoring the research approach to the cultural and linguistic context of the populations, particularly if the participants can express themselves in different languages within the same thread of discussion. Another implication is that the conversational agent must be built to accommodate these languages. Something that is still technically challenging in AI research and at the risk of inadvertently introducing biases that could reduce controllability and affect the reliability of the results.

Finally, there exist inherent systematic limitations in all endeavors to enact profound societal transformations in any country. Using “virtual” conversational agents is insufficient to emancipate oppressed Afghan women or address complex issues such as gender equality. The problem is multifaceted and deeply rooted in cultural, social, economic, and political factors. A technological solution, such as conversational agents, cannot solve it alone. The challenges faced by Afghan women require systemic changes in various aspects of society, such as access to education, legal protection, political representation, economic empowerment, awareness and advocacy, media representation, etc. However, some of these aspects could benefit from AI-enabled platforms targeting younger and ICT-literate generations that can mediate decisions that affect the local communities and instigate tangible social change^6^. It is essential to approach the challenge with a balanced perspective, acknowledging its potential benefits without overlooking the considerable societal barriers. Further research and a holistic approach that involves various social actors and mediation strategies will be necessary to determine the real impact of such technological interventions on gender equality in the region.

### Challenges

We now explore some of the challenges that this line of research is set to overcome in order to drive meaningful advancements in the realm of gender equality and AI. Several aspects of conversational AI need to be discussed before advocating its use to address gender inequality or try to “empower” anyone. The first challenge comes with the choice of the data used to train the Machine Learning models that the agents use^39^. There are always risks that the textual datasets used to train such models are loaded with stereotypical concepts of gender, and the results could easily perpetuate this bias^67^. Such stereotypes could either be misogynistic or simply inappropriate for the target population. This instance brings us to the second challenge, the design choices when building AI-powered online platforms, given that there could be different interpretations of inclusion, equality, and gender biases^14^. Such choices often follow cultural styles that might not be appropriate for certain populations or minorities. For example, western cultural standards are predominantly adopted in deliberation research^14^. Different cultures have distinct conversational styles and interaction modes between genders^68,69^. Designing conversational AI must therefore account for gender behaviors, cultural diversities, communication styles, and social norms^70^. The third challenge is overcoming the trustability stigmata that assigns to conversational agents, or “chatbots”, various malicious activities^71–73^. There is qualitative progress in the area, with realistic, quasi-meaningful conversational systems such as ChatGPT^19^, but the impacts of such large language models (LLMs) are still scrutinized^74,75^. Chatbots could cause reinforcement that might lead to echo chambers or cascading effects that cause certain discussions to be insulated from others^76^. These points raise the problem of whether people should entrust their social interactions to conversational agents or not. Establishing trust between humans and artificial agents could be paramount to their social acceptance. These trustability issues led, for instance, to establish a global regulatory landscape for AI that sets core ethical principles for developing Trustworthy AI^37^.

## Conclusion

This study delves into some of the most pressing issues of our time: the rapid advancement of AI technologies, the persistent gender inequality and discrimination against women, and the political turmoil in some regions of the world. By exploring the potential of conversational AI to amplify women's presence and agency on social networks, the study addresses the need for innovative solutions to promote gender equality in online spaces. The findings shed light on the distinct ways female and male debaters engage in online discussions, paving the way for a deeper understanding of gender dynamics in virtual interactions. Moreover, introducing a conversational agent demonstrated its potential to influence the contributions of participants, highlighting the agent's role in facilitating more inclusive and enriched conversations. By addressing the gender inequality that persists in societies like Afghanistan^77^, where women face significant barriers, this research highlights the potential of technology to act as a positive force for change^20^.

This work is not without limitations. First, it is important to generalize the scope of applicability beyond the specific context of Afghanistan or Afghan women. Second, the growing introduction of autonomous and intelligent agents to the online sphere will undoubtedly change how human agency is defined^12,64^. It is therefore crucial to remain mindful of ethical considerations surrounding this shift particularly when dealing with sensitive topics such as gender inequality. Future research should continue to address these challenges to ensure that AI technologies are ethically harnessed^37^.

### Supplementary Information

Supplementary Information.

## Data Availability

The datasets generated and analyzed in this study are available from the corresponding author upon request.

## Code availability

The computer code used to generate the results reported in the manuscript is available to editors and reviewers upon request.
