# Supplementary material for: Conversational agents enhance women's contribution in online debates
Source: Sci Rep. 2023 Sep 4;13:14534. doi: 10.1038/s41598-023-41703-3 (PMC10477209; doi:10.1038/s41598-023-41703-3)

Conversational Agents Enhance Women's Contribution in Online Debates

Rafik Hadfi[1*], Shun Okuhara[2], Jawad Haqbeen[1], Sofia Sahab[1], Susumu Ohnuma[3], and Takayuki Ito[1]
[1] Department of Social Informatics, Kyoto University, Kyoto, Japan
[2] Graduate School of Engineering, Mie University, Mie, Japan
[3] Department of Behavioral Science, Hokkaido University, Sapporo, Japan
[*] rafik.hadfi@i.kyoto-u.ac.jp

Supplementary Information (S1)

Conversational AI: Architecture, Interface, and Discussion Topic

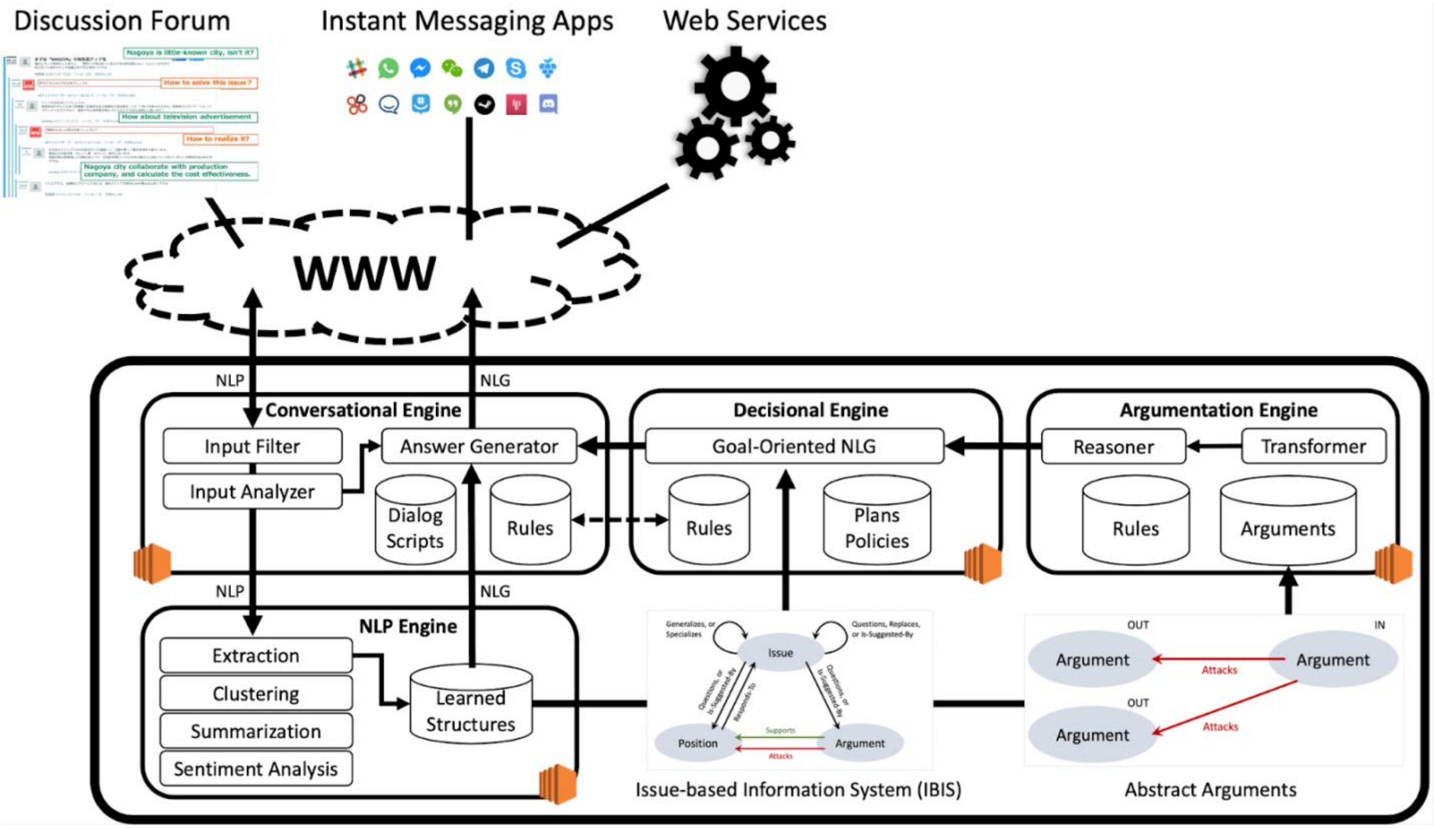

S1.1. The architecture of the conversational agent used in the study.

The conversational agent in S1.1 relies on Natural Language Processing (NLP) techniques to extract, process and produce argumentative text. First, a *Conversational Engine* processes the utterances from the raw textual content of the discussion and filters out the harmful and non-textual content. Then, an *NLP Engine* extracts the textual constituents of the discussion and classifies them into issues (or questions), positions (or ideas), or arguments (pros and cons) according to the issue-based information system (IBIS). The classifier relies on an Artificial Neural Network (ANN) called Bidirectional Long Short-Term Memory (Bi-LSTM) to classify the textual content into IBIS categories. The conversational agent must be able to identify the argumentative content of the discussion to facilitate the debates. To this end, the agent transforms any IBIS representation into an

argumentation graph and reasons about its elements using an *Argumentation Engine*. Once the argumentative content is processed, a *Decisional Engine* applies specific rules to generate new utterances. These facilitation rules could, for instance, encourage the participants to brainstorm, debate, or deliberate. These rules could also specify which subsets of the participants should the conversational agent interact with. Finally, the *Conversational Engine* will generate the final messages using Natural Language Generation (NLG) techniques and post them to the active discussion thread at a designated position.

The conversational agent was previously evaluated in terms of its performance in mining arguments and the satisfaction of the participants in the discussions facilitated by the agent. The classifier was trained from real-world English discussion data and yielded an accuracy of over 80% F value for IBIS utterances' classification and 90% for predicting the associations between these utterances. The conversational agent was also evaluated in terms of the participants' satisfaction after interacting with the agent in online discussions. We have shown that the participants' satisfaction in the discussions facilitated by the agent is more than average compared to cases where there was no facilitation.

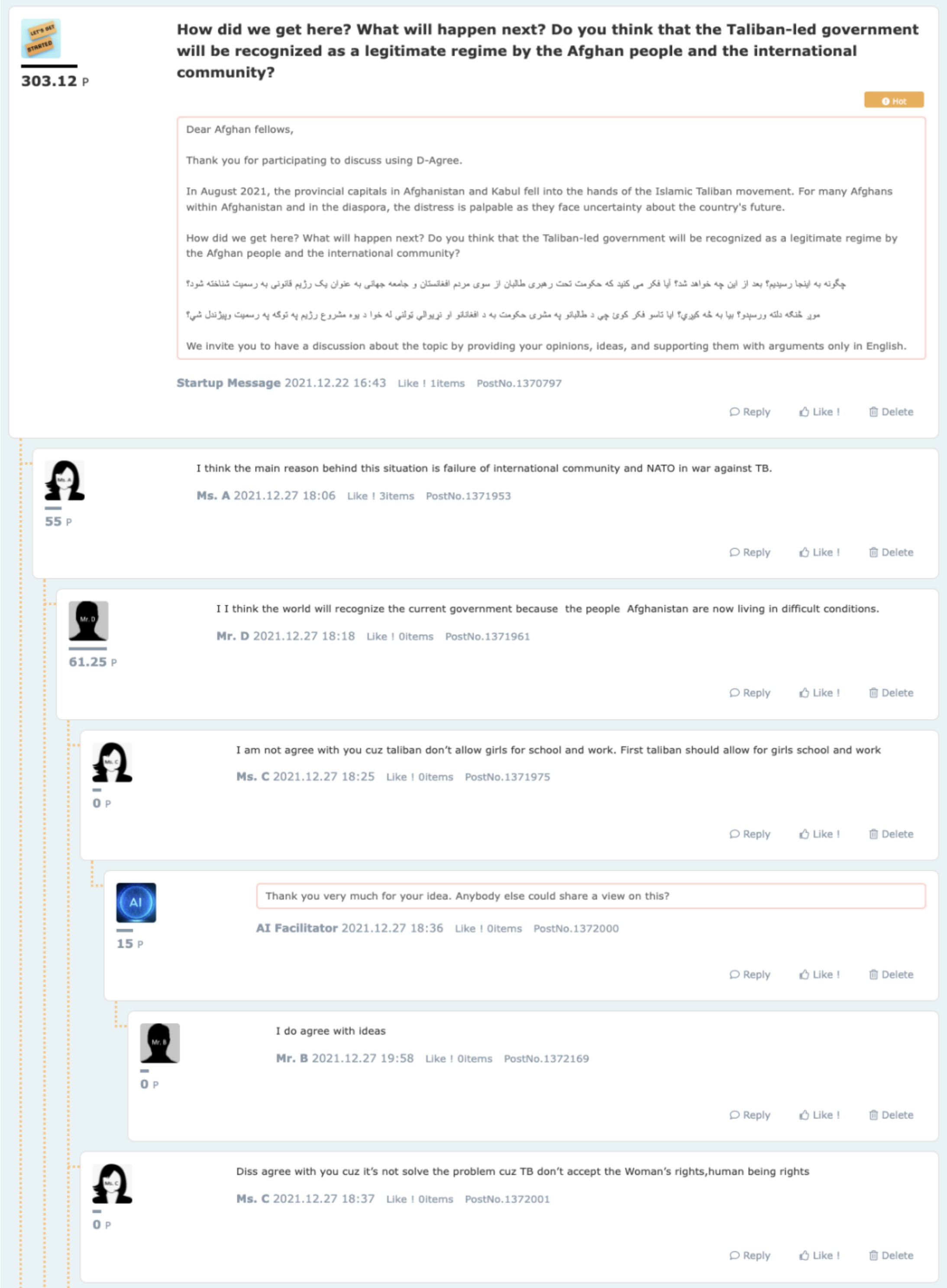

S1.2. The startup message sets the topic of the discussion at the top of each discussion thread. The message is posted in English and Persian for all experimental groups. After the startup message is posted, the participants join their respective discussion spaces and start the discussions. In this mixed group discussion example, "Ms. A" is the first person to post a message by replying to the startup message. "Mr. D" replied to "Mr. A" by pressing the "Reply" button and writing his message. As the messages accumulate, the conversational agent, named here "AI Facilitator", starts replying to the participants based on predefined rules. In this case, the conversational agents replied to "Ms. C" with a supporting message. The replies reflect different patterns of interaction between the actors of the discussions. In this example, we distinguish one male-to-female interaction (symbolized by ▢→◯), two female-to-male interactions (symbolized by ◯→▢), one AI-to-female interaction (symbolized by △→◯), and one male-to-AI interaction (symbolized by ▢→△). Messages posted below the startup message (symbolized by a cross ✕) designate interactions that do not directly address a specific participant. In the example, this interaction (symbolized by ◯→ ✕) is due to the first message of "Ms. A".

Supplementary Information (S2)

Normality Tests

We performed several ANOVA tests to compare the means of the counts of the IBIS elements (Issues, ideas, pros, const) in group compositions ◯▢, △▢, ▢ and △◯▢ where the symbol ◯ represents female participants, ▢ represents male participants, and △ the conversational agent present in each discussion alongside the human participants. Before the ANOVA, we conducted normality tests to assess the data distribution. The boxplots, histograms, and quartiles are illustrated below.

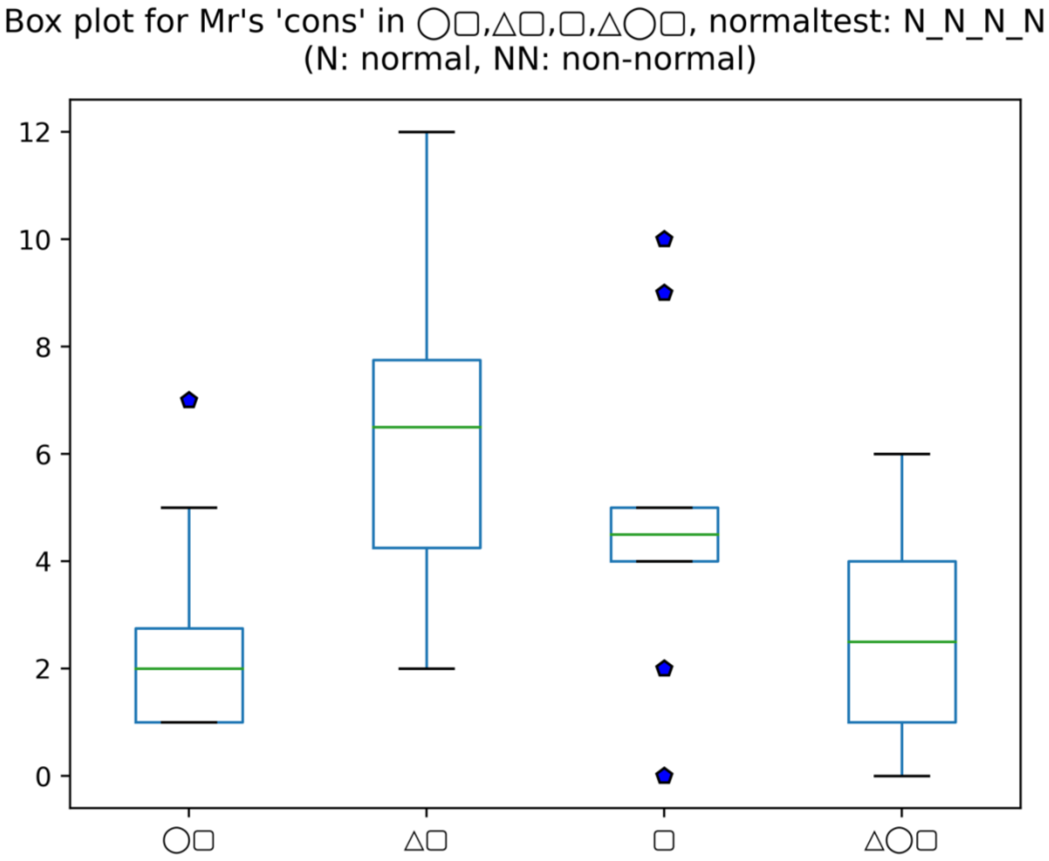
Box plot for Mr's 'cons' in ◯▢,△▢,▢,△◯▢, normaltest: N_N_N_N
(N: normal, NN: non-normal)
◯▢
△▢
▢
△◯▢

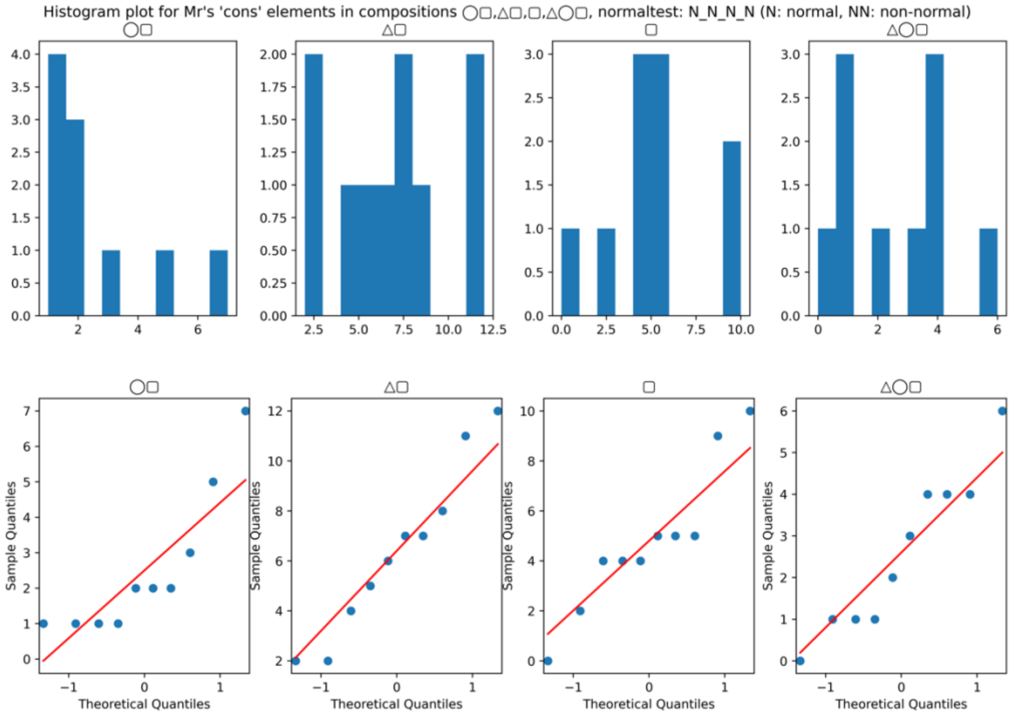
Histogram plot for Mr's 'cons' elements in compositions ◯▢,△▢,▢,△◯▢, normaltest: N_N_N_N (N: normal, NN: non-normal)
◯▢
△▢
▢
△◯▢
Sample Quantiles
Theoretical Quantiles

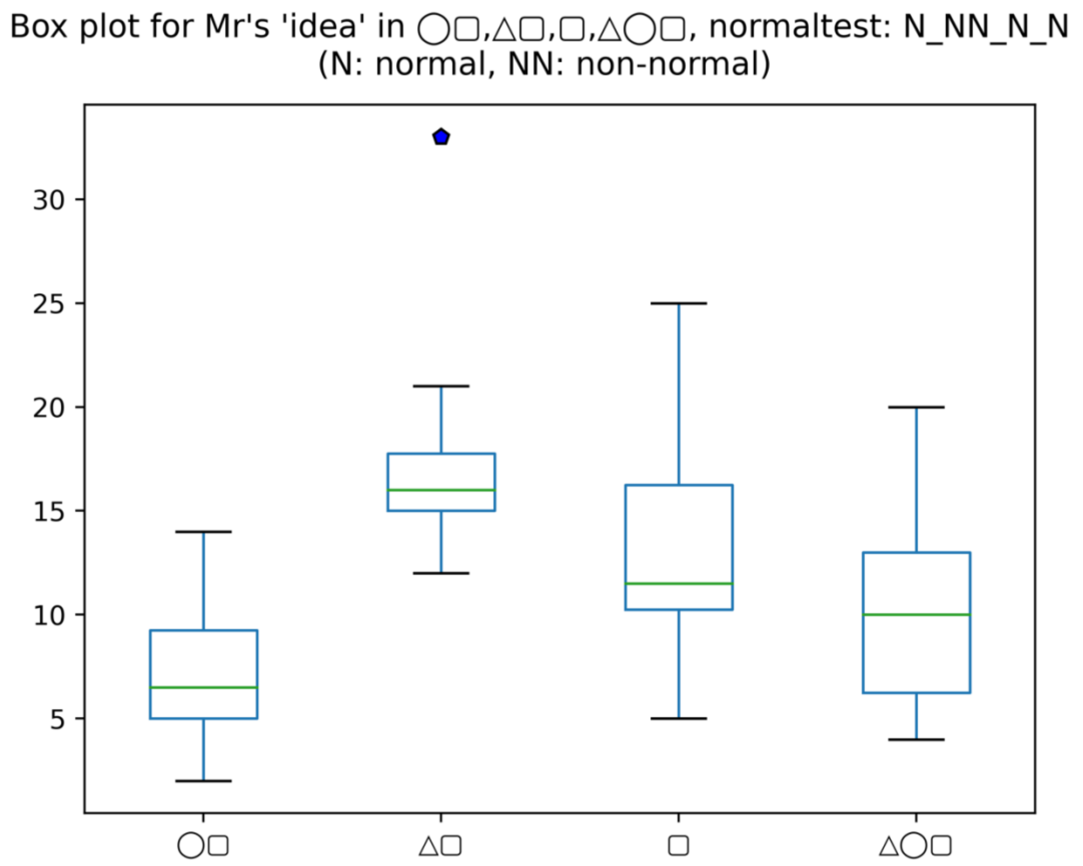
Box plot for Mr's 'idea' in ◯▢,△▢,▢,△◯▢, normaltest: N_NN_N_N
(N: normal, NN: non-normal)
30
25
20
15
10
5
◯▢
△▢
▢
△◯▢

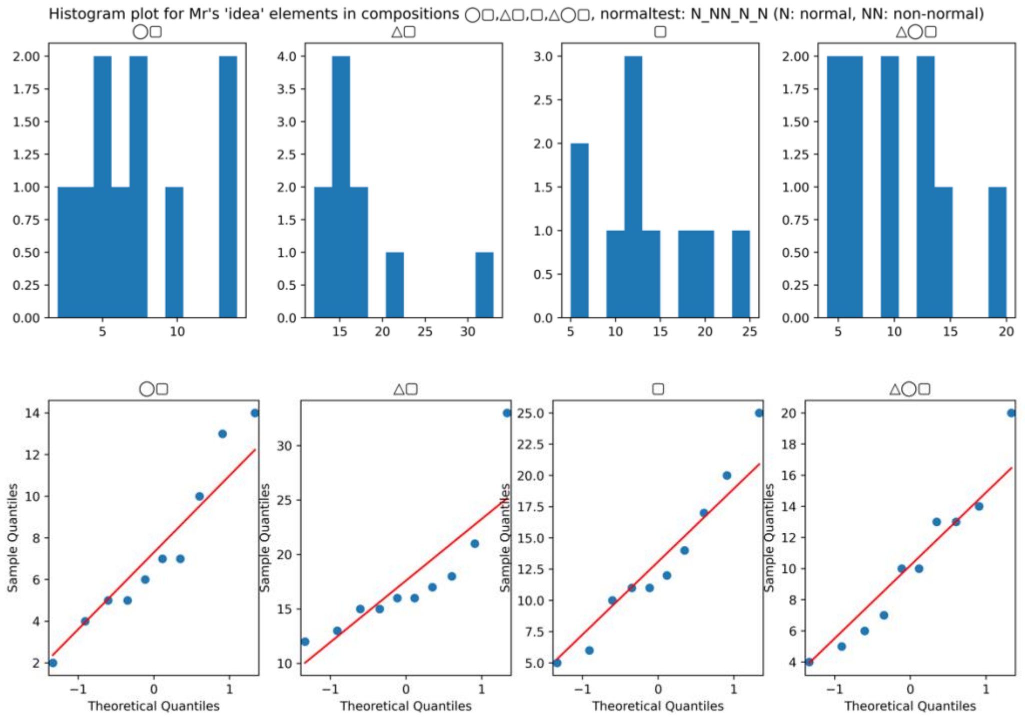
Histogram plot for Mr's 'idea' elements in compositions ◯▢,△▢,▢,△◯▢, normaltest: N_NN_N_N (N: normal, NN: non-normal)
◯▢
△▢
▢
△◯▢
Sample Quantiles
Theoretical Quantiles

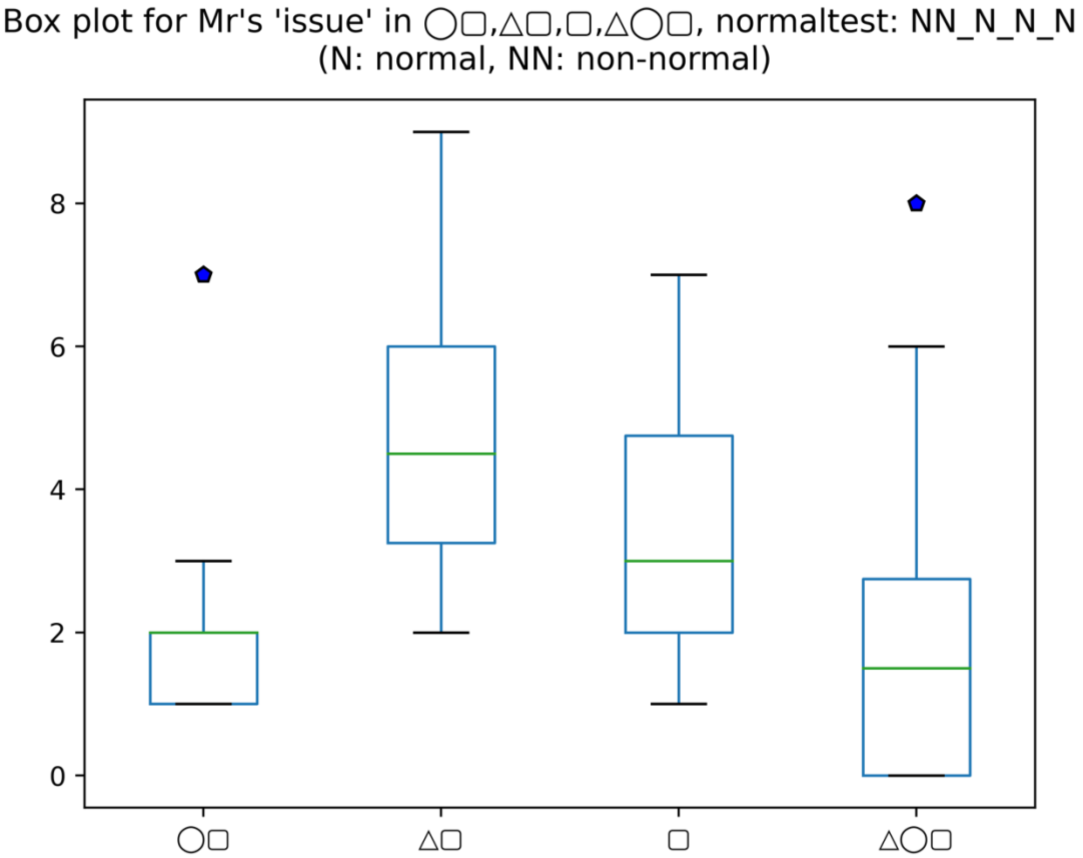
Box plot for Mr's 'issue' in ◯▢,△▢,▢,△◯▢, normaltest: NN_N_N_N
(N: normal, NN: non-normal)
◯▢
△▢
▢
△◯▢

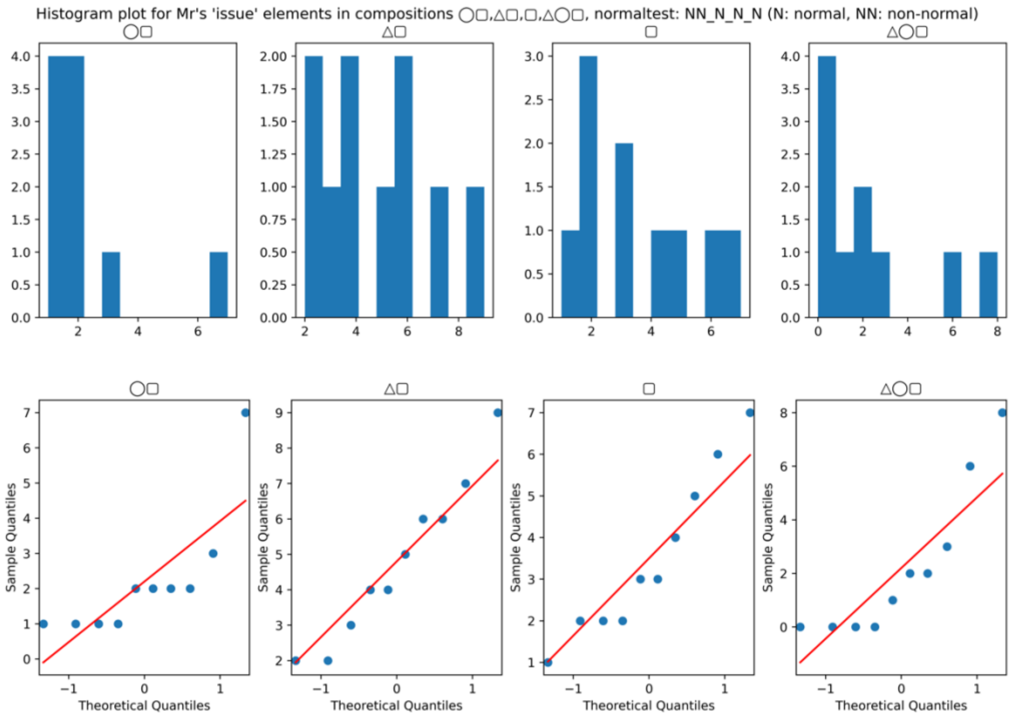
Histogram plot for Mr's 'issue' elements in compositions ◯▢,△▢,▢,△◯▢, normaltest: NN_N_N_N (N: normal, NN: non-normal)
◯▢
△▢
▢
△◯▢
Sample Quantiles
Theoretical Quantiles

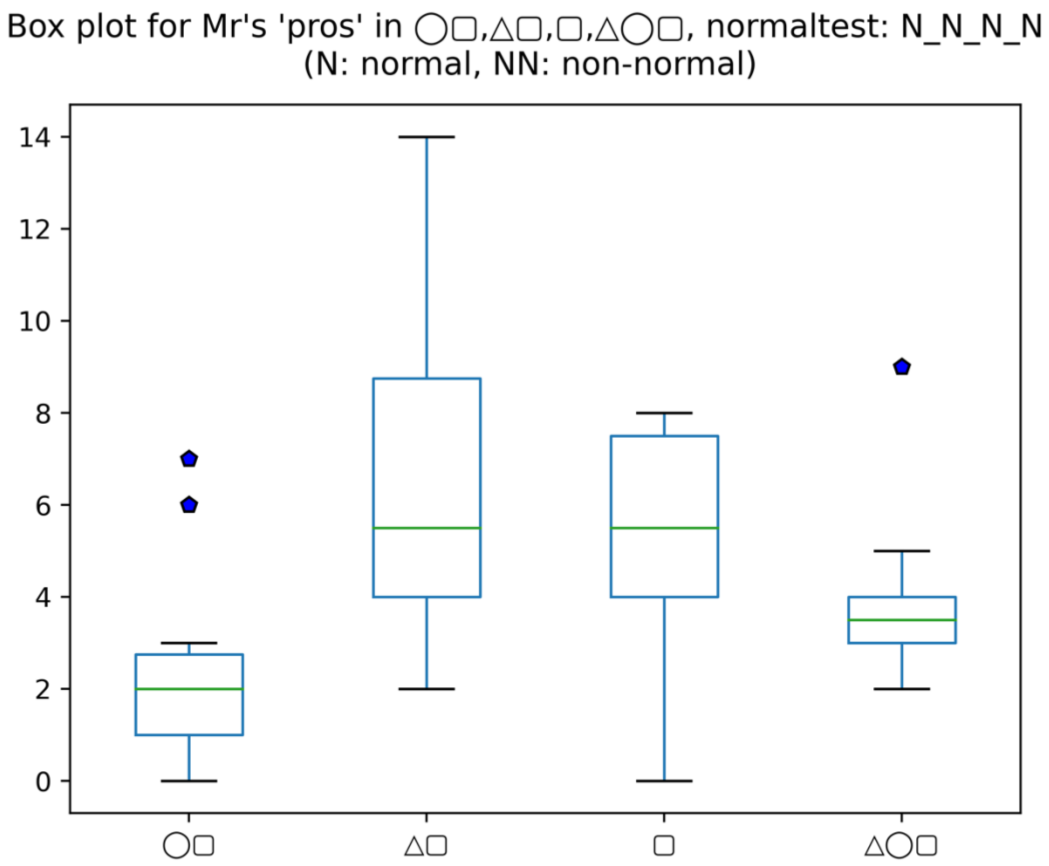
Box plot for Mr's 'pros' in ◯▢,△▢,▢,△◯▢, normaltest: N_N_N_N
(N: normal, NN: non-normal)
◯▢
△▢
▢
△◯▢

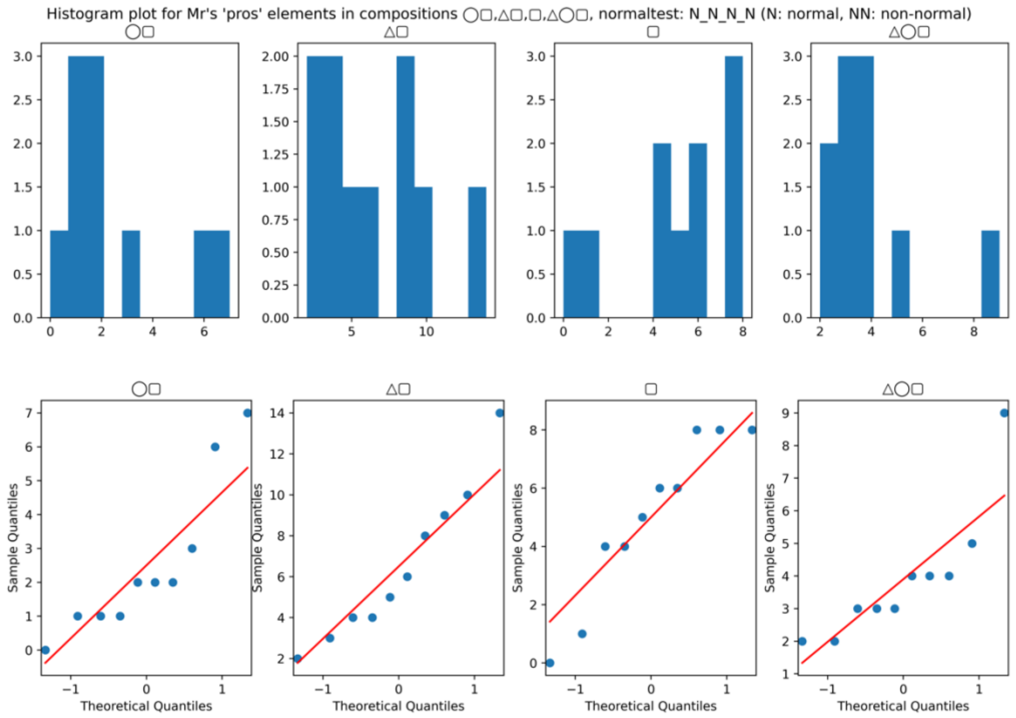
Histogram plot for Mr's 'pros' elements in compositions ◯▢,△▢,▢,△◯▢, normaltest: N_N_N_N (N: normal, NN: non-normal)
◯▢
△▢
▢
△◯▢
Sample Quantiles
Theoretical Quantiles

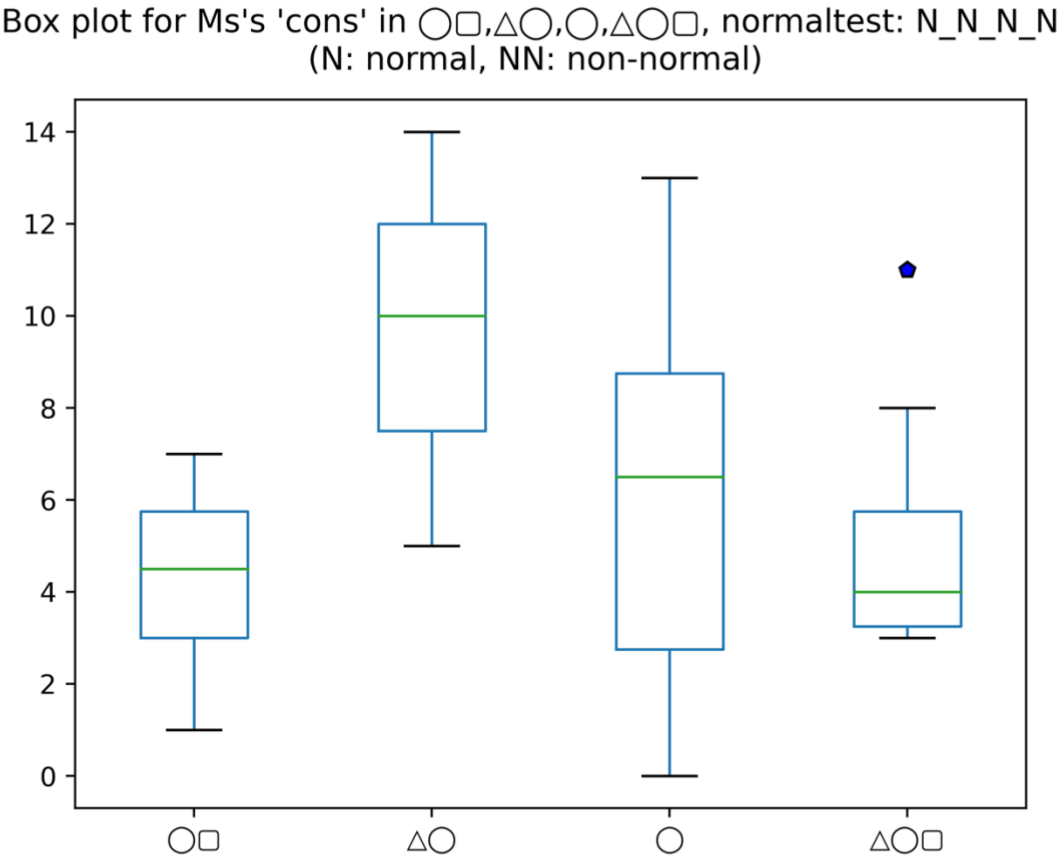
Box plot for Ms's 'cons' in ◯▢,△◯,◯,△◯▢, normaltest: N_N_N_N
(N: normal, NN: non-normal)
◯▢
△◯
◯
△◯▢

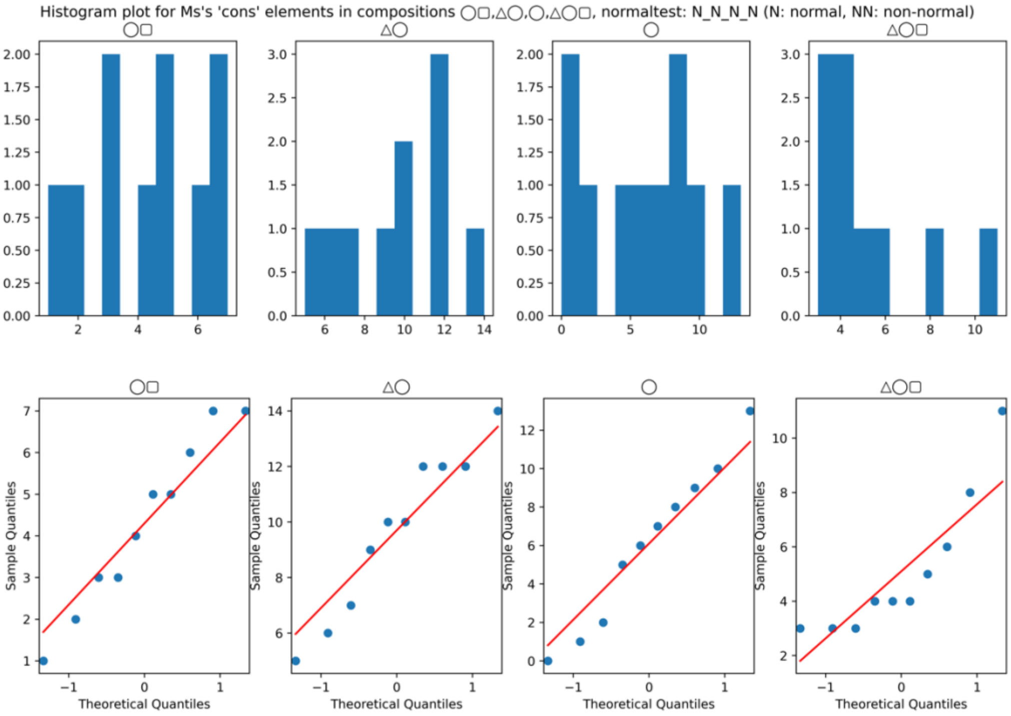
Histogram plot for Ms's 'cons' elements in compositions ◯▢,△◯,◯,△◯▢, normaltest: N_N_N_N (N: normal, NN: non-normal)
◯▢
△◯
◯
△◯▢
Sample Quantiles
Theoretical Quantiles

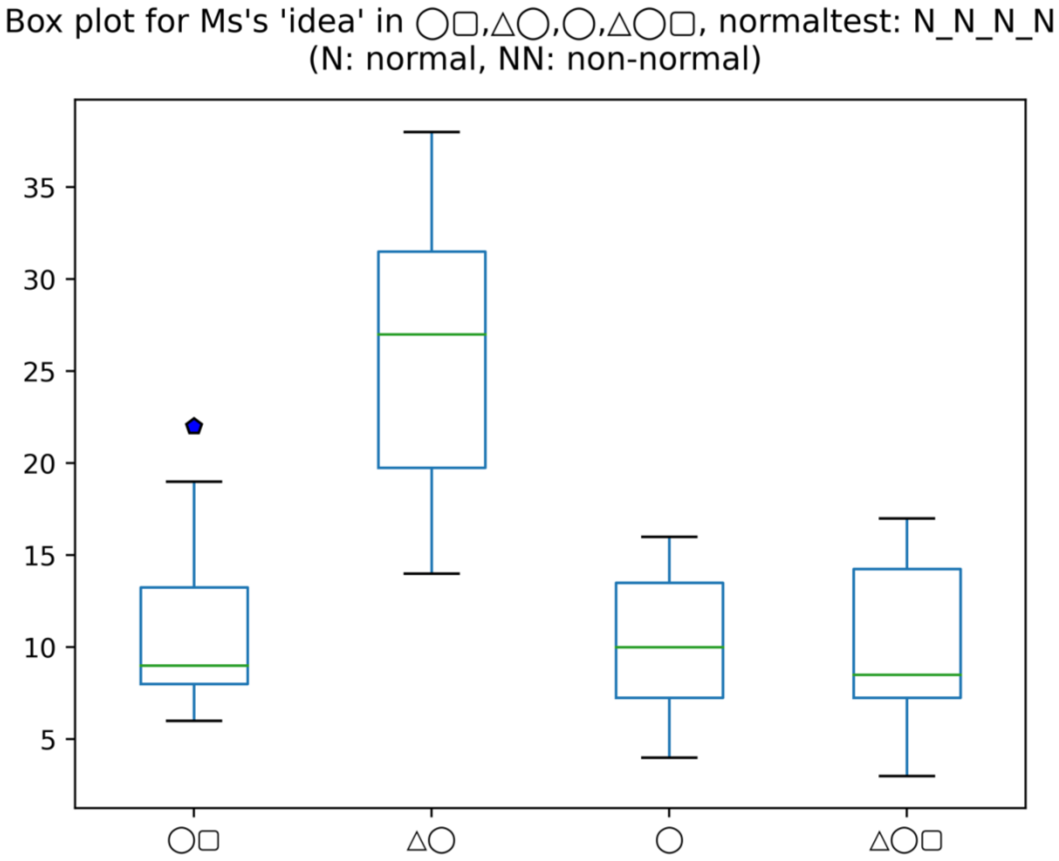
Box plot for Ms's 'idea' in ◯▢,△◯,◯,△◯▢, normaltest: N_N_N_N
(N: normal, NN: non-normal)
5
10
15
20
25
30
35
◯▢
△◯
◯
△◯▢

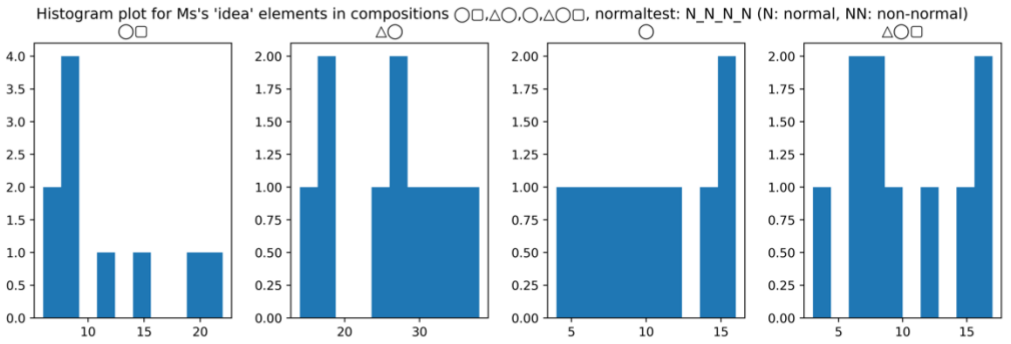
Histogram plot for Ms's 'idea' elements in compositions ◯▢,△◯,◯,△◯▢, normaltest: N_N_N_N (N: normal, NN: non-normal)
◯▢
△◯
◯
△◯▢

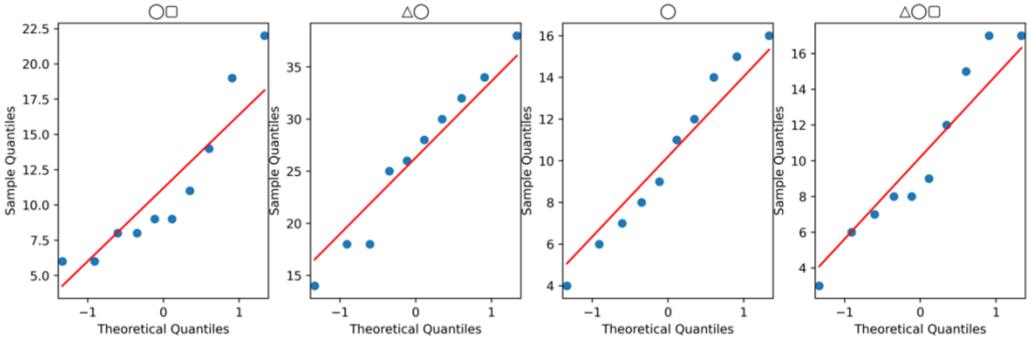
◯▢
△◯
◯
△◯▢
Sample Quantiles
Theoretical Quantiles

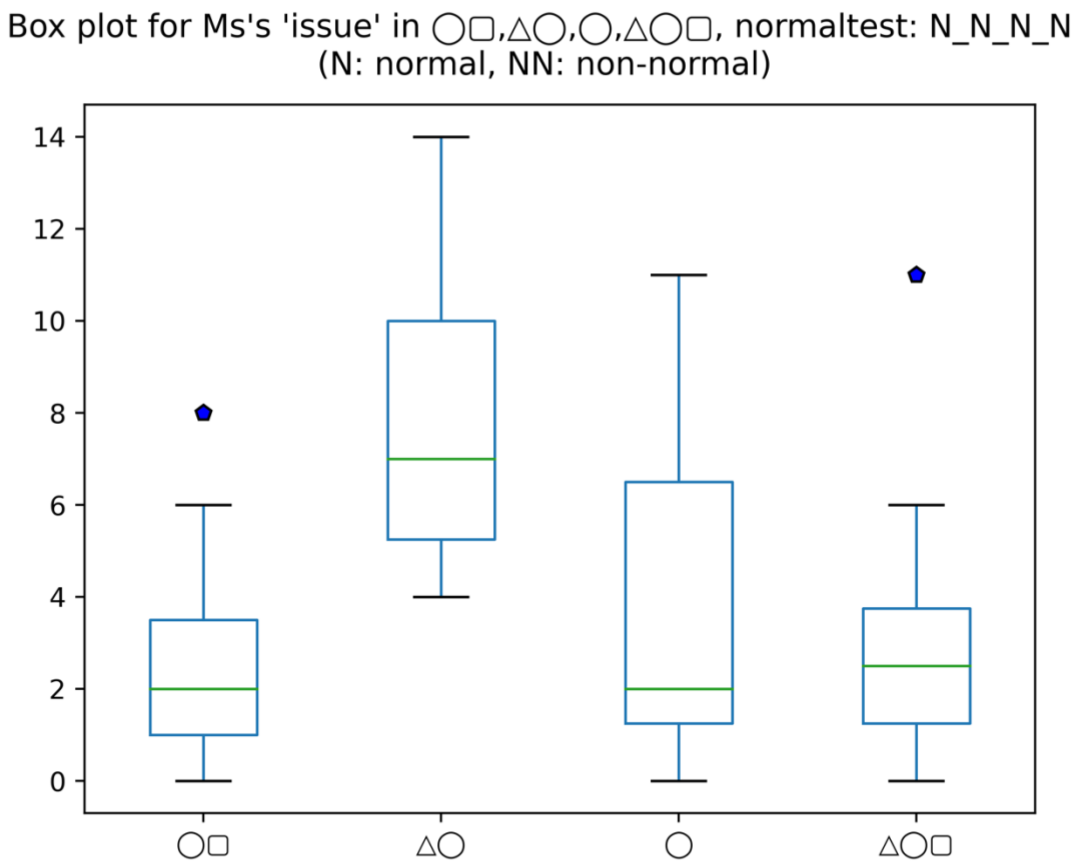

Box plot for Ms's 'issue' in ◯▢,△◯,◯,△◯▢, normaltest: N_N_N_N
(N: normal, NN: non-normal)
◯▢
△◯
◯
△◯▢

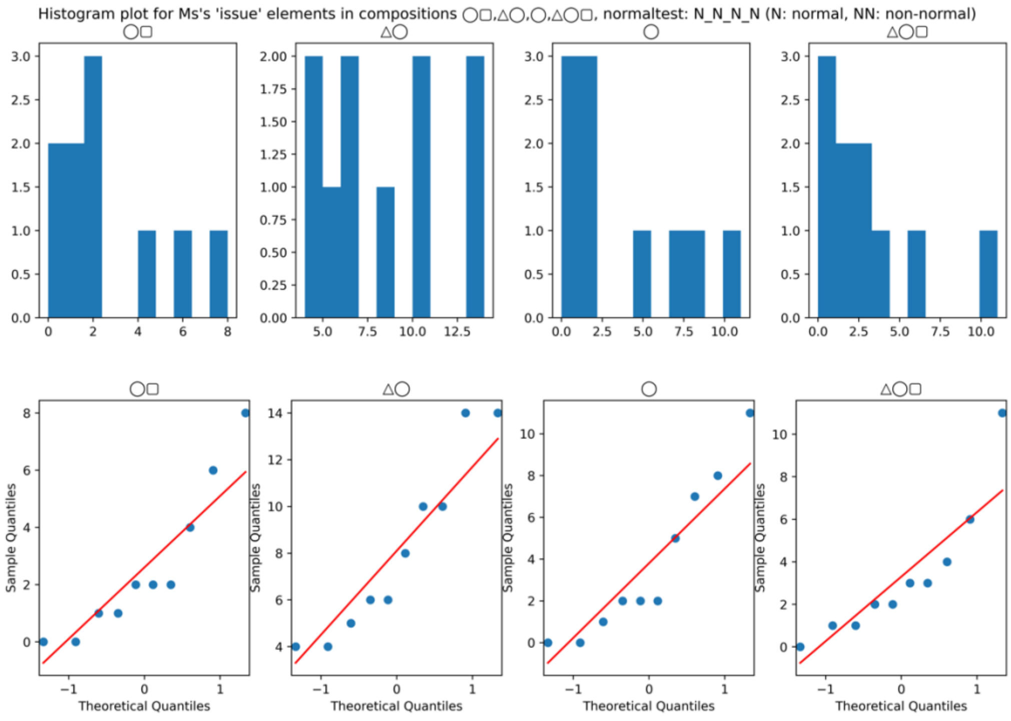

Histogram plot for Ms's 'issue' elements in compositions ◯▢,△◯,◯,△◯▢, normaltest: N_N_N_N (N: normal, NN: non-normal)
◯▢
△◯
◯
△◯▢
Sample Quantiles
Theoretical Quantiles

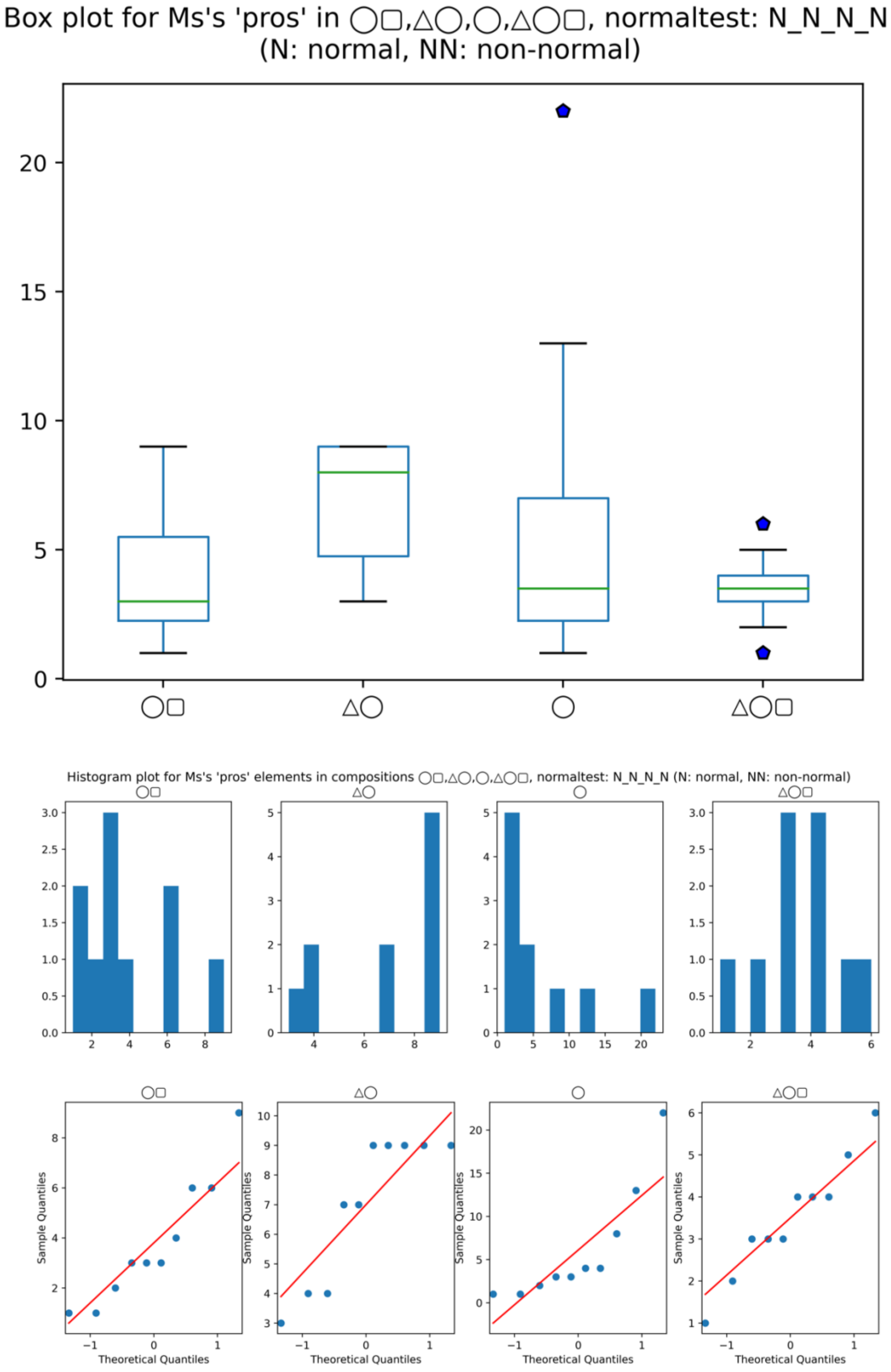
Box plot for Ms's 'pros' in ◯▢,△◯,◯,△◯▢, normaltest: N_N_N_N
(N: normal, NN: non-normal)
Histogram plot for Ms's 'pros' elements in compositions ◯▢,△◯,◯,△◯▢, normaltest: N_N_N_N (N: normal, NN: non-normal)
Sample Quantiles
Theoretical Quantiles

Supplementary Information (S3)

U Tests

Below are the U Tests for paired group compositions. For the description of the group compositions, we adopt a notation where the symbol ◯ represents female participants, ▢ represents male participants, and △ the conversational agent present in each discussion alongside the human participants.

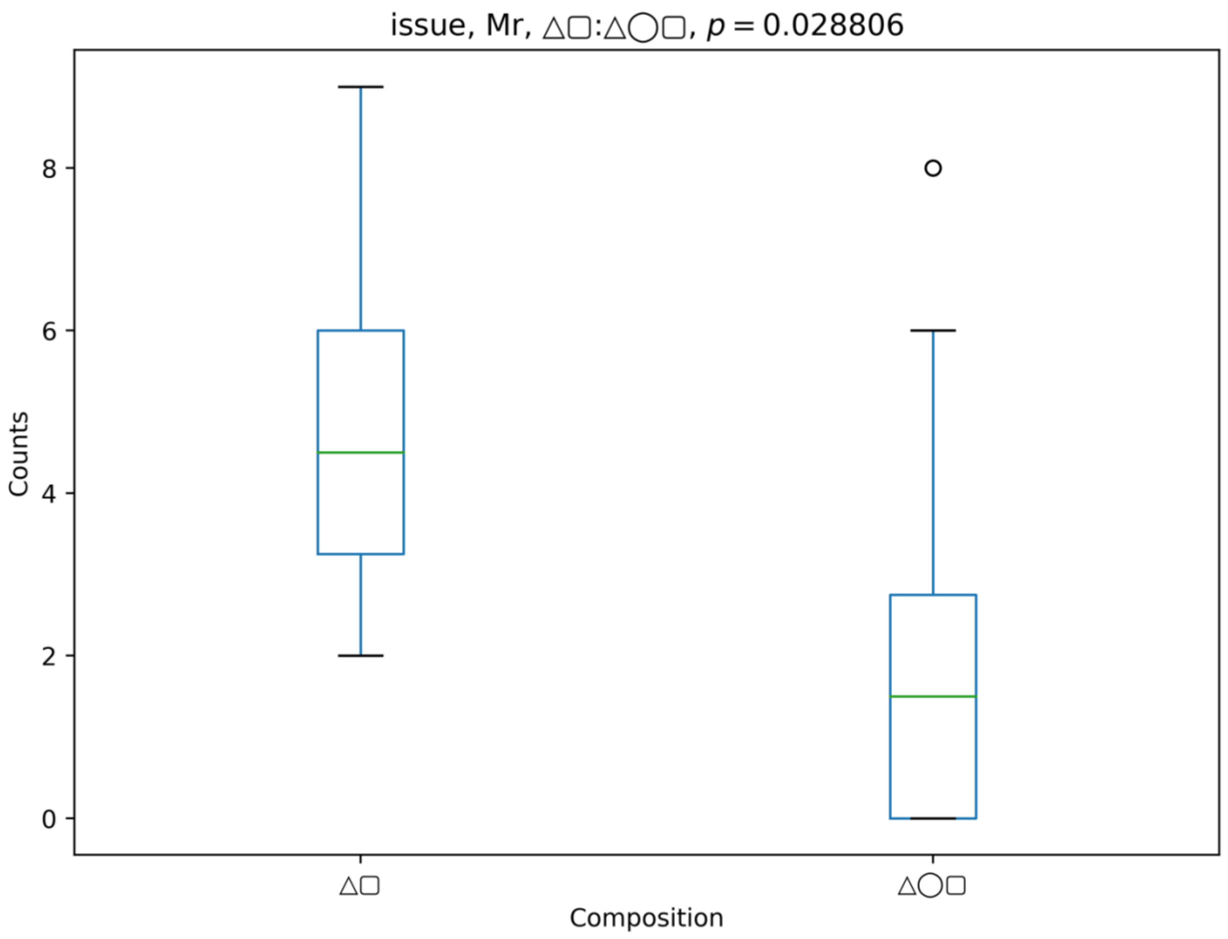

issue, Mr, △▢:△◯▢, p = 0.028806
Counts
0
2
4
6
8
△▢
△◯▢
Composition

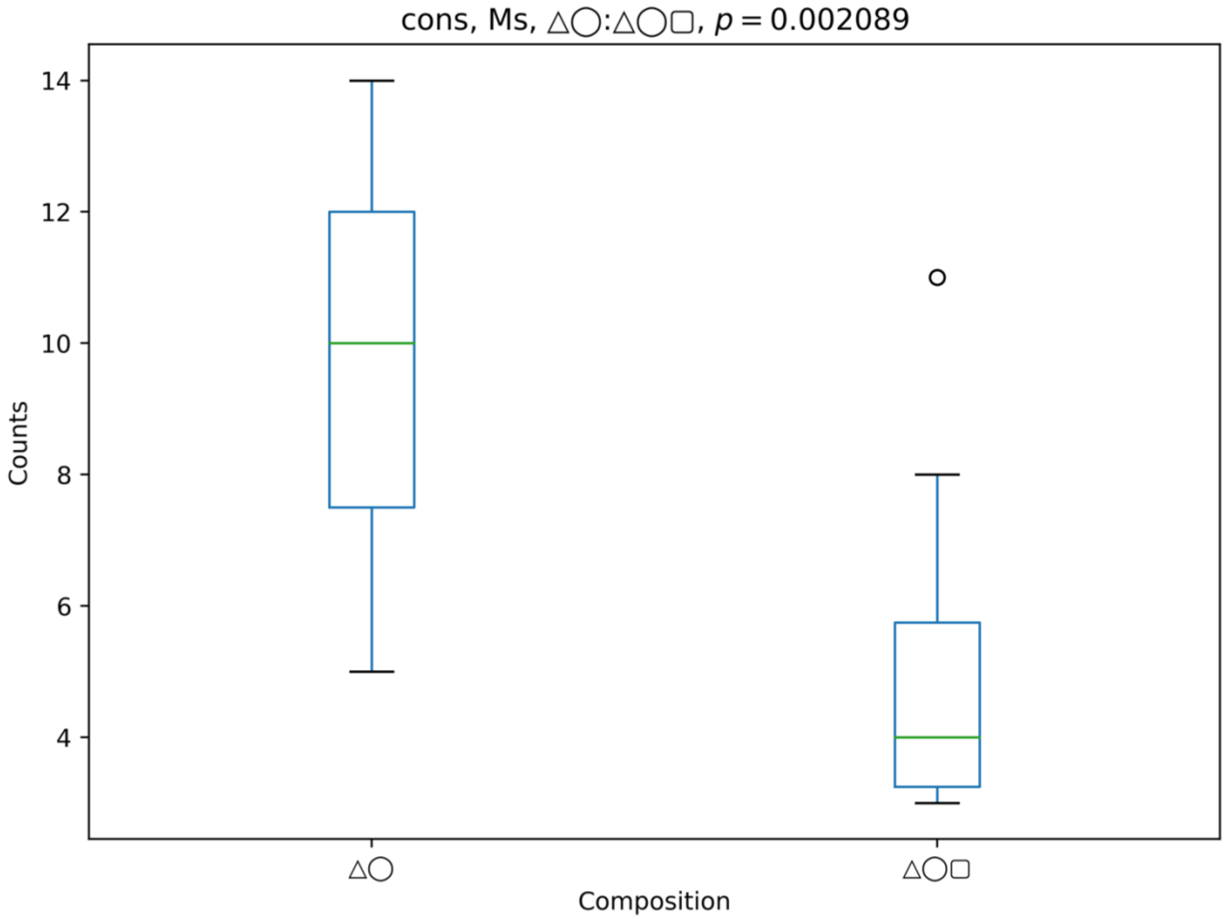
cons, Ms, △◯:△◯▢, p = 0.002089
Counts
14
12
10
8
6
4
△◯
△◯▢
Composition

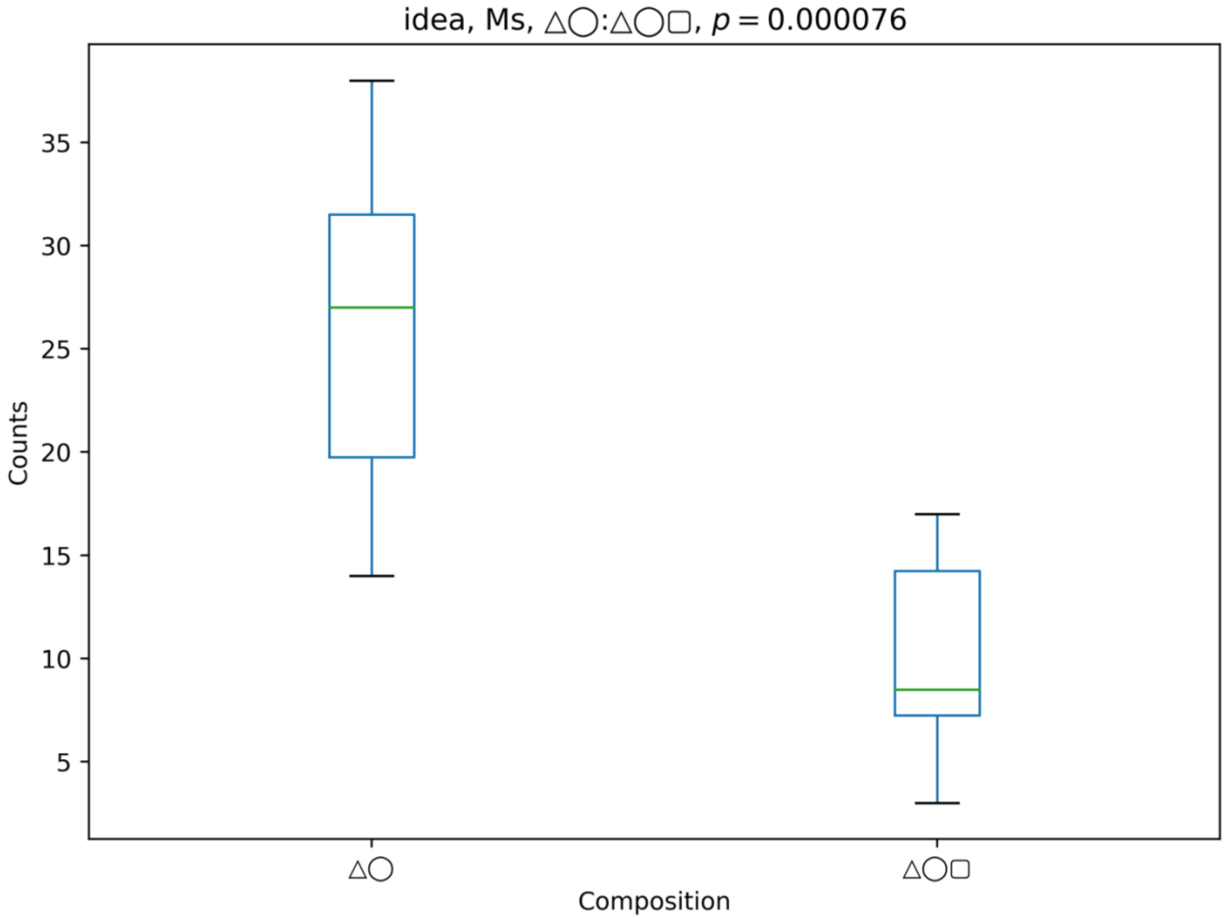

idea, Ms, △◯:△◯▢, $p = 0.000076$
35
30
25
20
15
10
5
Counts
△◯
△◯▢
Composition

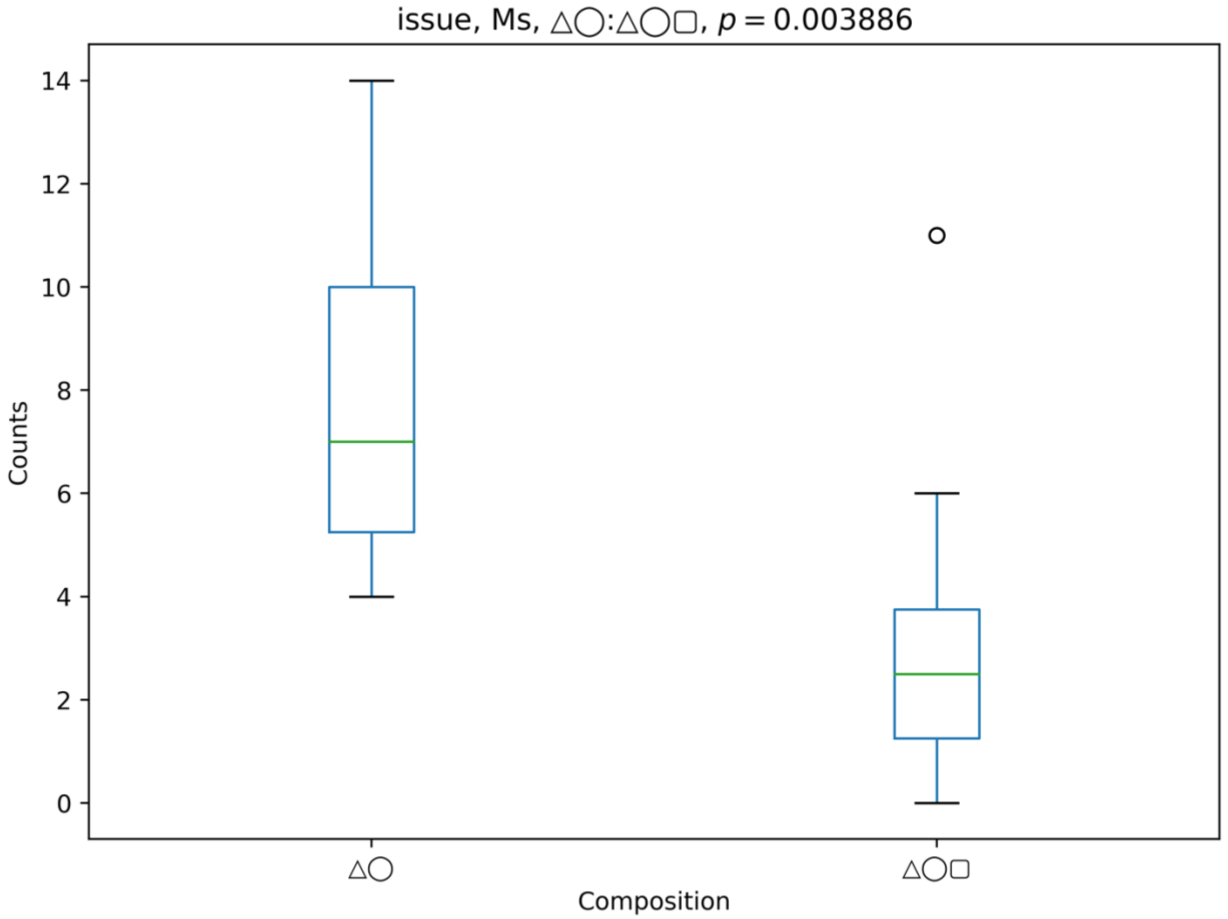
issue, Ms, △◯:△◯▢, $p = 0.003886$
Counts
0
2
4
6
8
10
12
14
△◯
△◯▢
Composition

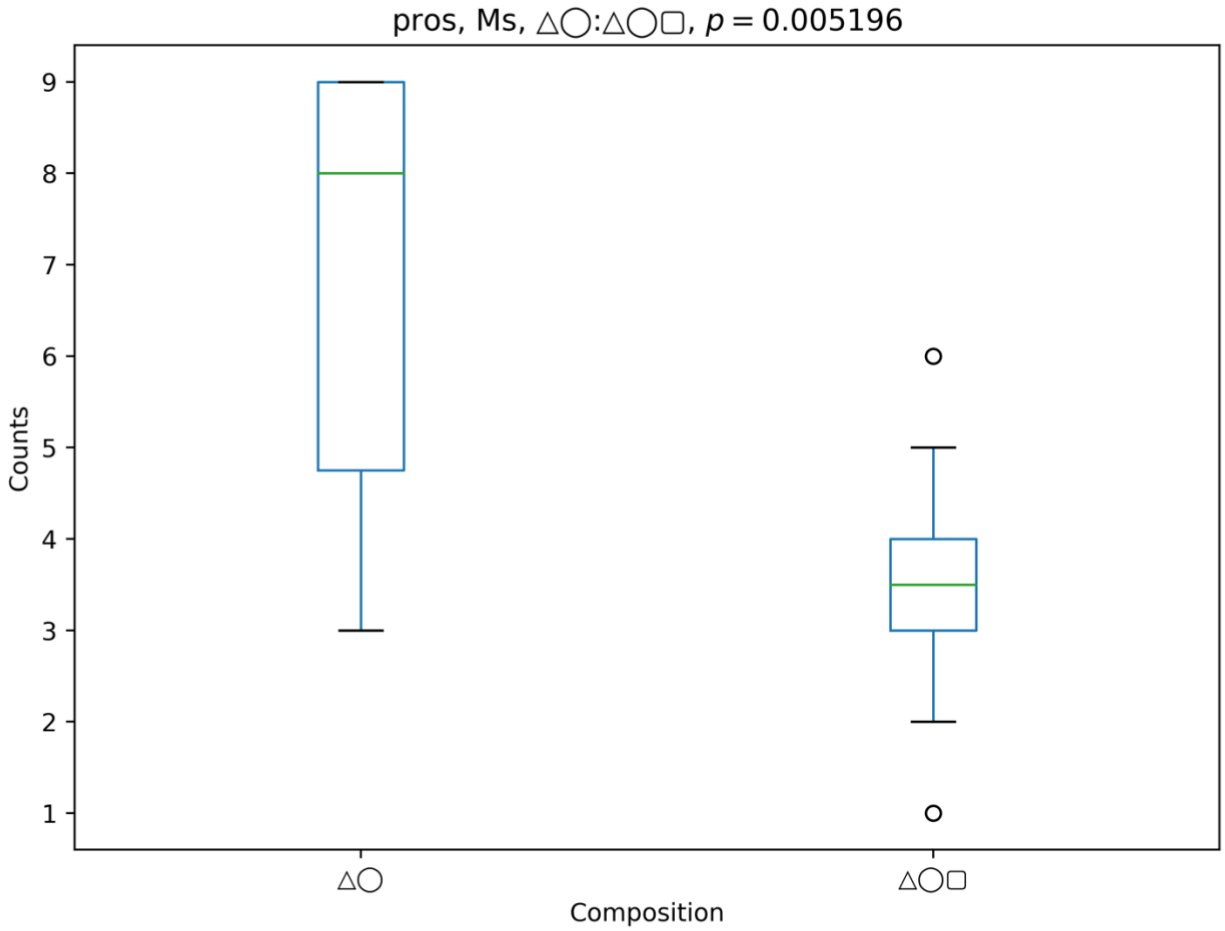

pros, Ms, △◯:△◯▢, p = 0.005196
Counts
9
8
7
6
5
4
3
2
1
△◯
△◯▢
Composition

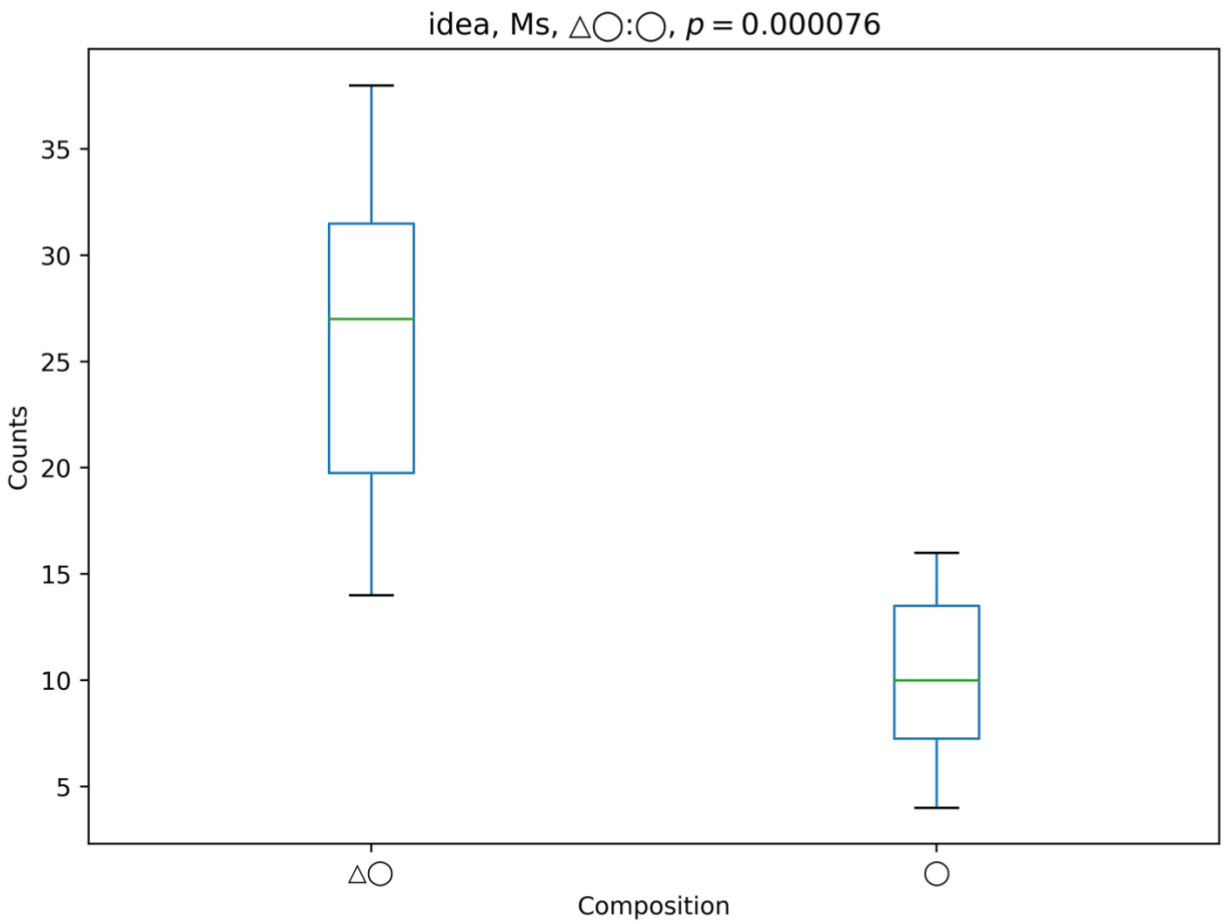
idea, Ms, △◯:◯, $p = 0.000076$
Counts
35
30
25
20
15
10
5
△◯
◯
Composition

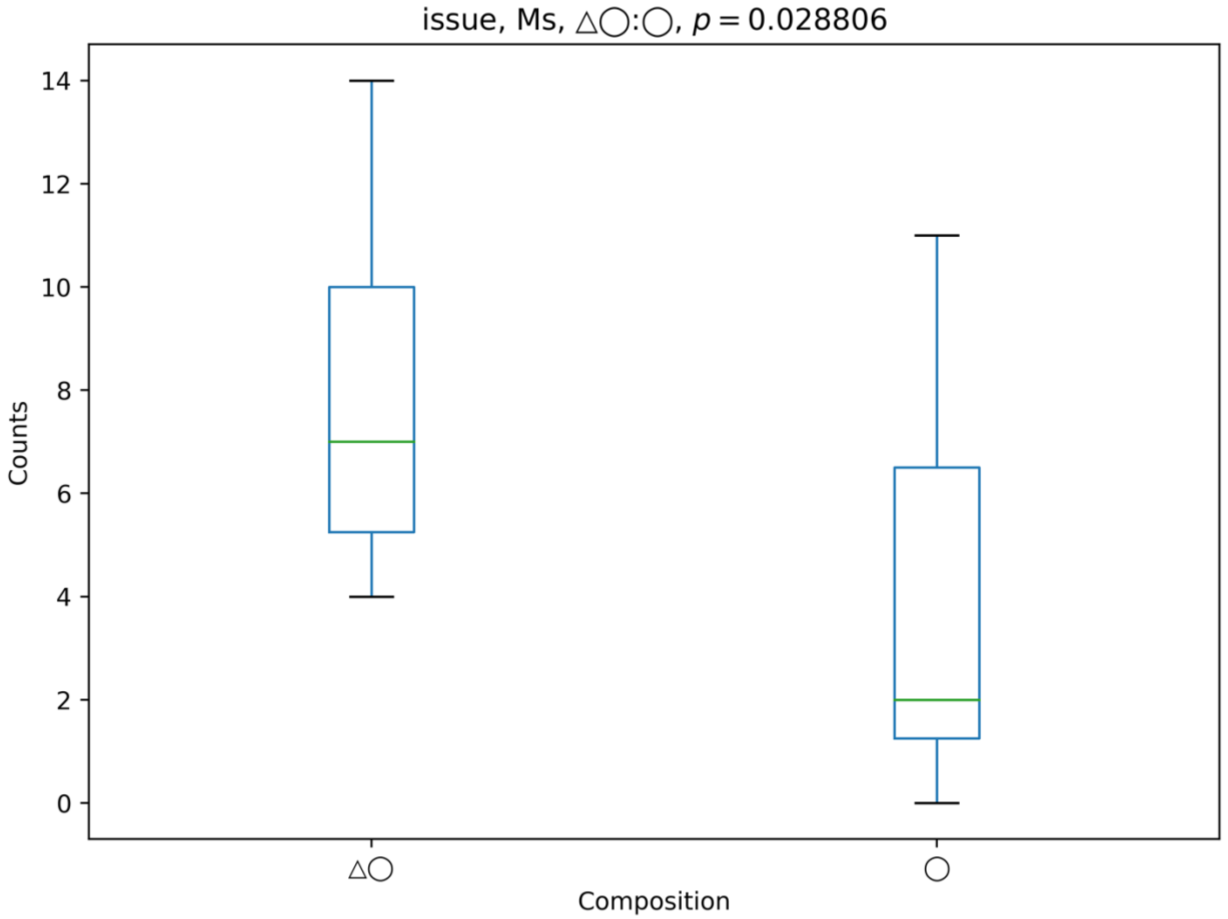

issue, Ms, △◯:◯, p = 0.028806
Counts
14
12
10
8
6
4
2
0
△◯
◯
Composition

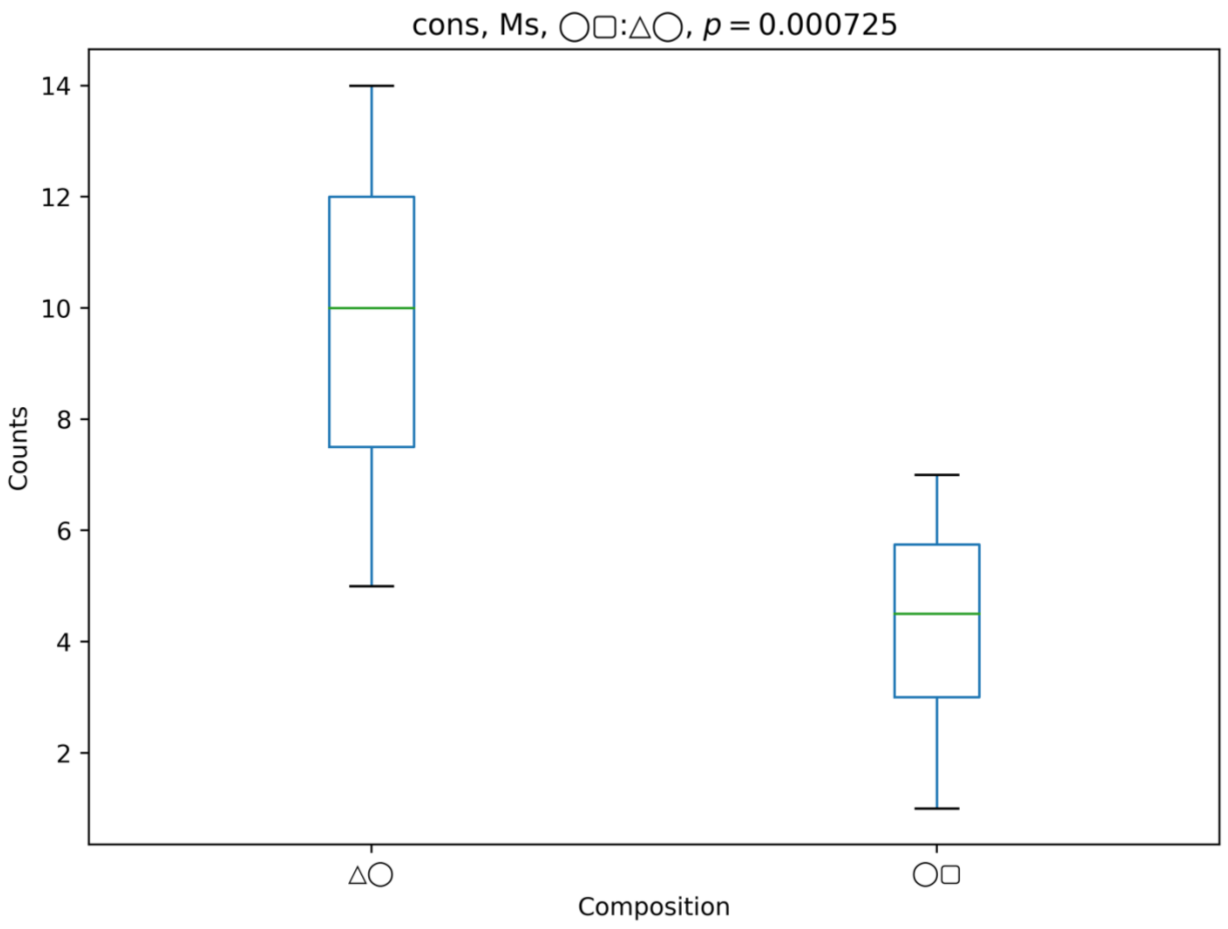

cons, Ms, ◯▢:△◯, $p = 0.000725$
Counts
14
12
10
8
6
4
2
△◯
◯▢
Composition

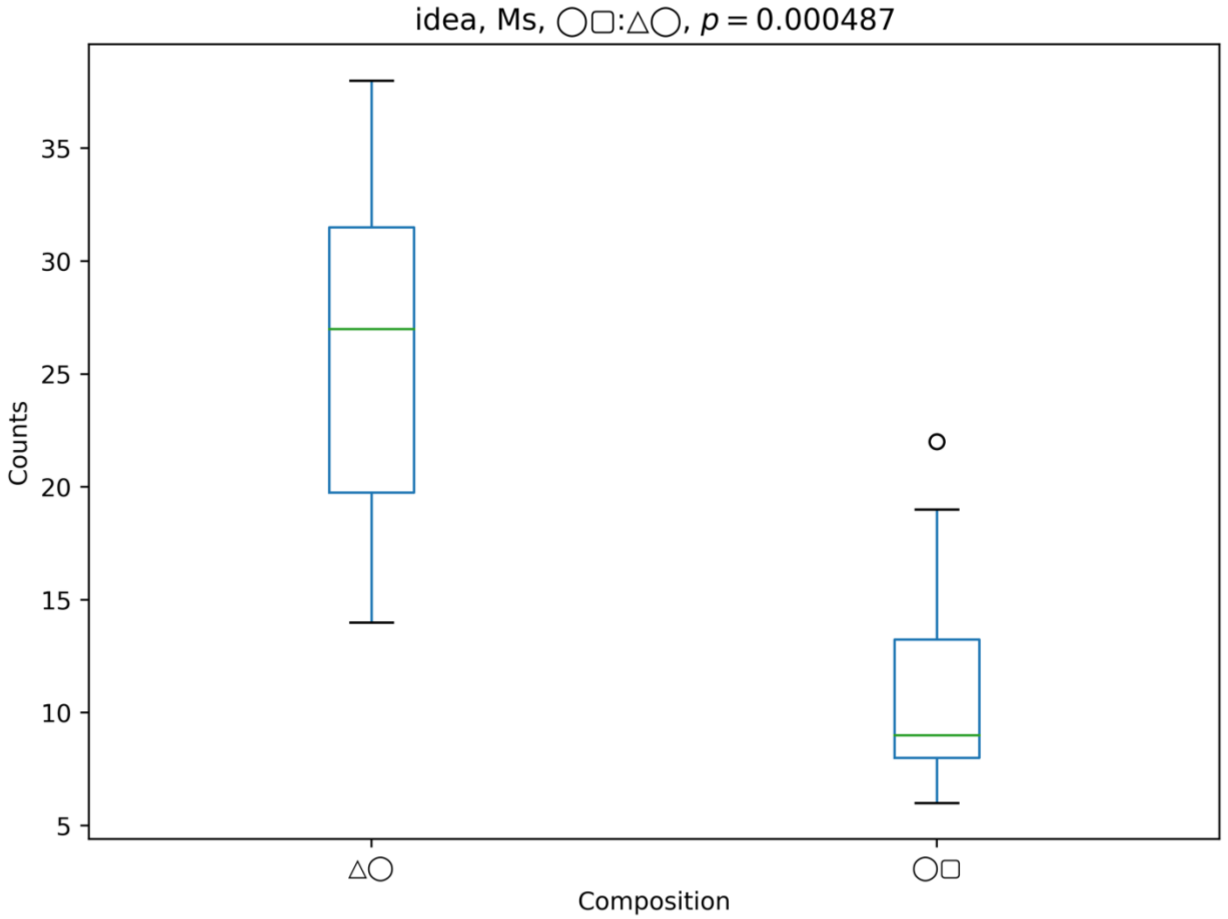

idea, Ms, ◯▢:△◯, $p = 0.000487$
Counts
35
30
25
20
15
10
5
△◯
◯▢
Composition

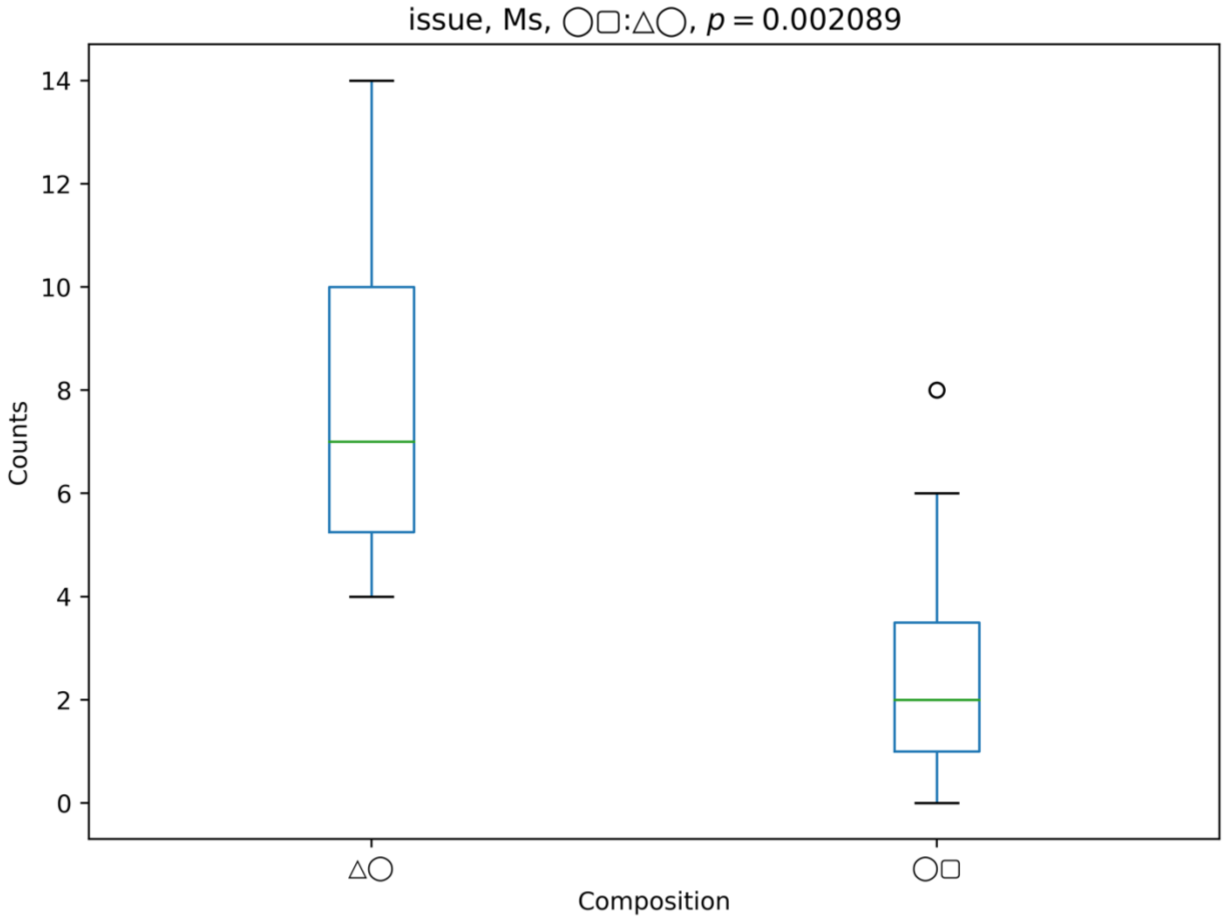

issue, Ms, ◯▢:△◯, $p = 0.002089$
Counts
14
12
10
8
6
4
2
0
△◯
◯▢
Composition

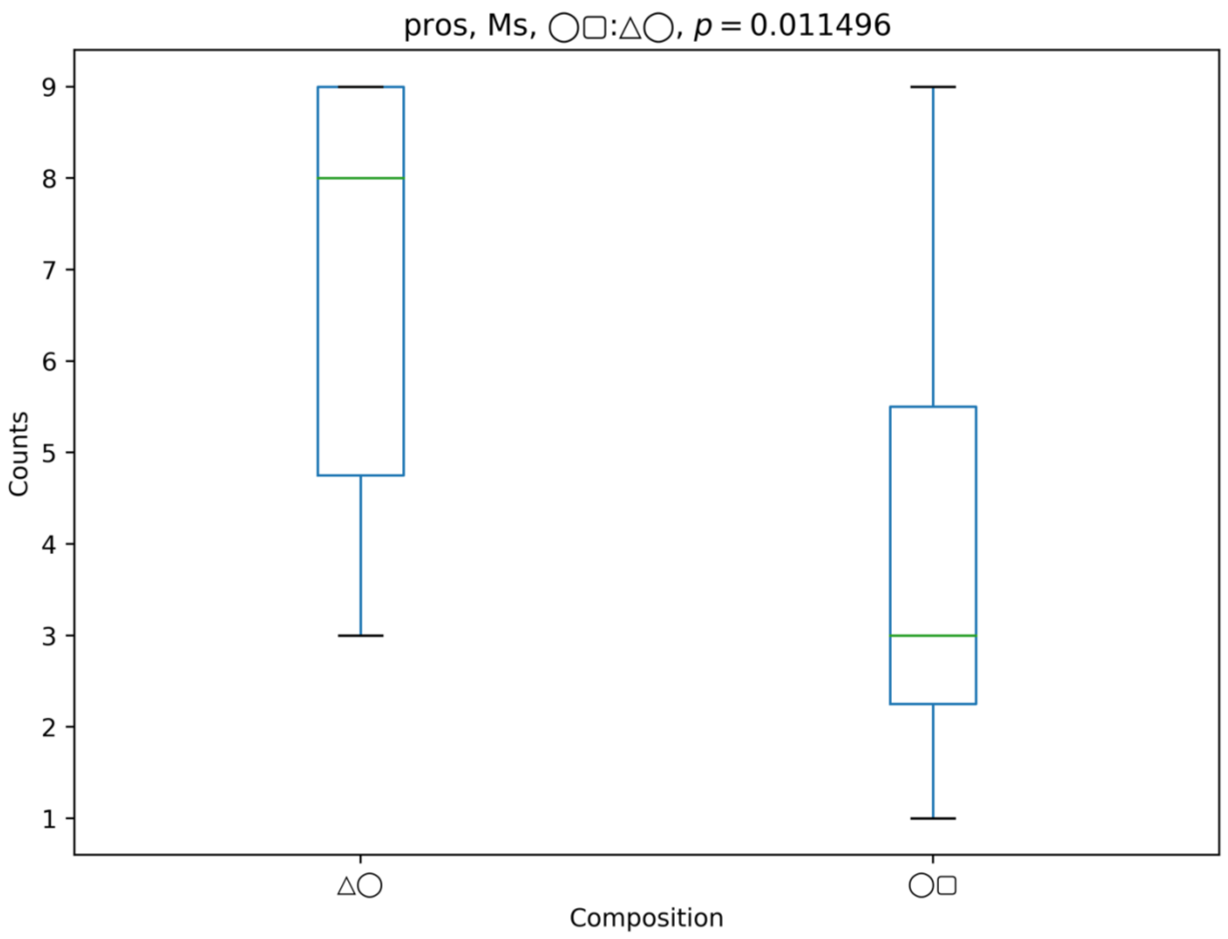

pros, Ms, ◯▢:△◯, $p = 0.011496$
Counts
9
8
7
6
5
4
3
2
1
△◯
◯▢
Composition

Qualified Subjects (S4)

Subjects' distribution

Out of the n=10000 participants that answered the call on Survey Monkey, n=5898 were selected based on the criteria of age, education, English language level, formal English language testing, and the scores in the general knowledge questionnaires.

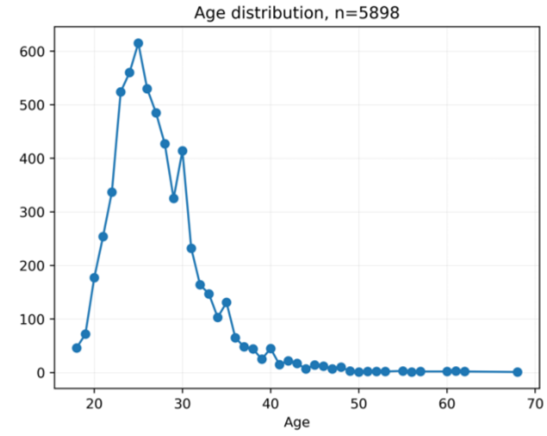

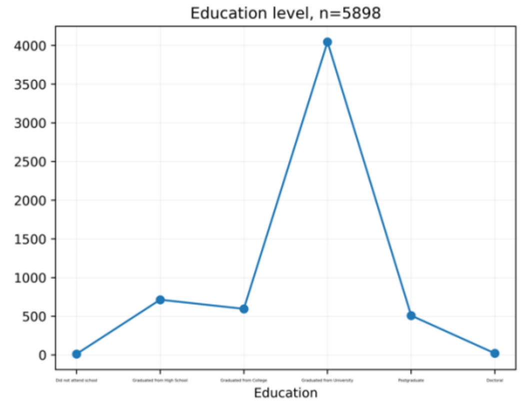

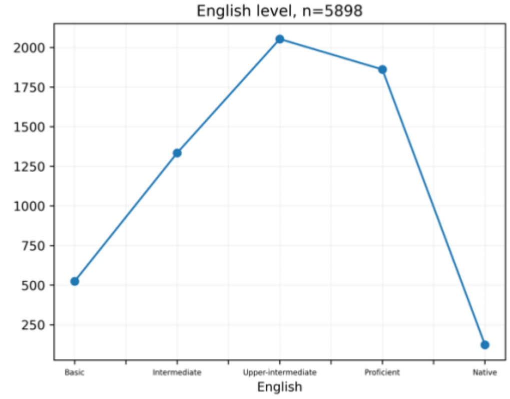

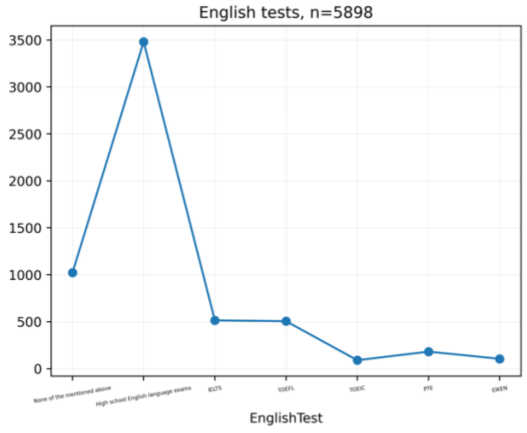

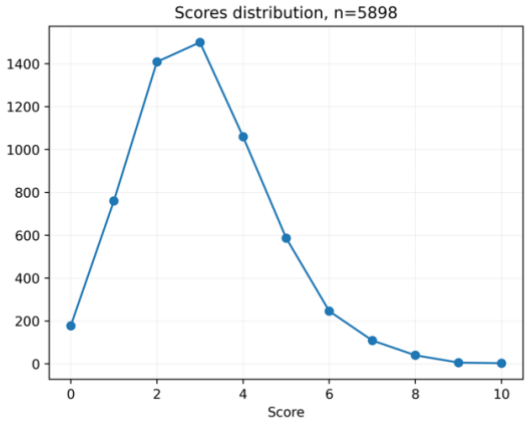

Supplement: Supplementary file 1 — Supplementary Information. [file 41598_2023_41703_MOESM1_ESM.pdf]
